# Antidiabetic Activity of Phenolic‐Rich Extracts From *Cistus albidus* (L.) in Alloxan‐Induced Diabetic Rats: *In Vitro*, *In Vivo*, and *In Silico* Investigations

**DOI:** 10.1002/fsn3.71658

**Published:** 2026-04-17

**Authors:** Aziz Zouhri, Naoual El Menyiy, Yahya El‐mernissi, Rafik El‐mernissi, Mohamed Reda Kachmar, Farhan Siddique, Rabie Kachkoul, Sumaira Nadeem, El Mouhri Ghita, Naima Mammate, Yousef A. Bin Jardan, Esmael M. Alyami, Mohammed Dauelbait, Mohammad Khalid, Lhoussain Hajji

**Affiliations:** ^1^ Bioactives and Environmental Health Laboratory, Faculty of Sciences Moulay Ismail University Meknes Morocco; ^2^ Laboratory of Pharmacology National Agency for Medicinal and Aromatic Plants Taounate Morocco; ^3^ Research Unit in Applied Chemistry, Faculty of Science and Techniques Abdelmalek Essaadi University Al Hoceima Morocco; ^4^ High Institute of Nursing Professions and Health Techniques Béni Mellal Morocco; ^5^ Valorization of Medicinal and Aromatic Plants and Environment, Faculty of Sciences Moulay Ismail University Meknes Morocco; ^6^ School of Pharmaceutical Science and Technology Tianjin University Tianjin P. R. China; ^7^ Faculty of Medicine and Pharmacy, Laboratory of Biochemistry Sidi Mohammed Ben Abdellah University Fez Morocco; ^8^ Higher Institute of Nursing Professions and Health Techniques Fez Morocco; ^9^ Department of Pharmaceutical Chemistry, Faculty of Pharmacy Bahauddin Zakariya University Multan Pakistan; ^10^ Laboratory of Epidemiology and Research in Health Sciences, Faculty of Medicine, Pharmacy, and Dental Medicine Sidi Mohammed Ben Abdellah University of Fez Fez Morocco; ^11^ Department of Pharmaceutics, College of Pharmacy King Saud University Riyadh Saudi Arabia; ^12^ Department of Biology, College of Science King Khalid University Abha Asir Saudi Arabia; ^13^ Research Centre for Advanced Materials Science (RCAMS) King Khalid University Abha Saudi Arabia; ^14^ University of Juba Juba South Sudan; ^15^ Department of Pharmaceutics, College of Pharmacy King Khalid University Abha Asir Saudi Arabia

**Keywords:** *α*‐amylase alloxan, antidiabetic activity, *Cistus albidus*, glibenclamide

## Abstract

Diabetes, a prevalent metabolic disorder, has prompted extensive research into natural remedies with hypoglycemic potential and minimal side effects. The aim of this study was to examine phenolic compounds using high‐performance liquid chromatography (HPLC). Acute toxicity was assessed in mice following OECD 423 criteria, and anti‐diabetic potential was tested by intraperitoneal injection of alloxan (150 mg/kg) to induce experimental diabetes. Acute toxicity assessment found no adverse effects, while chemical analysis revealed various phenolic compounds like rutin, quercetin, and gallic acid, known for their therapeutic properties. In vitro experiments with the aqueous extract showed significant inhibition of pancreatic *α*‐amylase enzyme activity (IC_50_ value: 119.813 ± 3.827 μg/mL). In vivo studies demonstrated the extract's efficacy in mitigating body weight loss, promoting hypoglycemic effects, and improving oral glucose tolerance. Additionally, the extract regulated biochemical parameters including total cholesterol, triglycerides, liver enzymes, and renal markers. These findings suggest that *Cistus albidus* leaf polyphenols possess anti‐hyperglycemic and α‐amylase inhibitory properties, supporting its traditional use in diabetes treatment and potential incorporation into future antidiabetic medications. Molecular docking analysis further supported the interaction of extract constituents with relevant target proteins, offering insights into potential therapeutic interventions.

## Graphical abstract

The phenolic‐rich aqueous extract of *Cistus albidus* leaves (CAAE) demonstrated significant antihyperglycemic activity in alloxan‐induced diabetic rats through improved glucose tolerance and modulation of metabolic biomarkers. CAAE also exerted lipid‐lowering and hepatoprotective effects, alongside notable α‐amylase inhibitory activity supported by molecular docking analysis. These findings highlight the multitarget therapeutic potential of CAAE as a promising natural strategy for diabetes management.

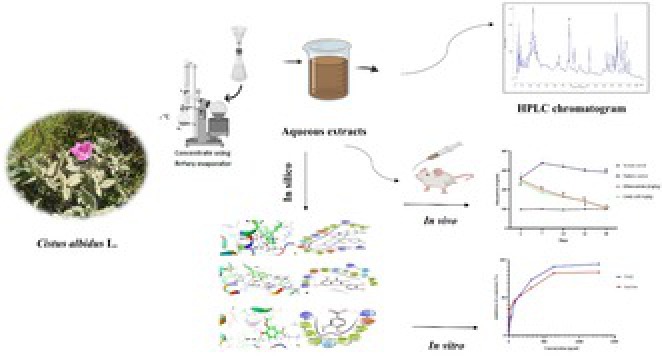

## 1 Introduction

In recent years, there has been a growing interest in harnessing the therapeutic potential of medicinal plants for combating diabetes (Sunil et al. 2012; Sabir et al. 2025), a complex metabolic disorder characterized by chronic hyperglycemia. Diabetes mellitus, often referred to as diabetes, is a global health concern affecting millions of people worldwide (Wild et al. 2004). The condition is associated with various complications, including cardiovascular disorders, neuropathy, nephropathy, and retinopathy (Umar et al. 2010), which significantly impact the quality of life and life expectancy of individuals living with diabetes.

Faced with this health challenge, medicinal plants have become a valuable resource in the search for new and effective treatments for diabetes. Traditional systems of medicine, such as in Morocco and other ancient systems around the world, have long recognized the potential of specific plant species in the management of diabetes. These plants are known to contain bioactive compounds with anti‐diabetic properties, ranging from blood sugar regulation to the protection of vital organs affected by diabetes.

*Cistus albidus* L., known as “Bouchikh”, has traditionally been a hypoglycemic and hypotensive agent against skin infections, respiratory infections, and intestinal pain (El‐Mernissi et al. 2023). Pharmacological studies have also demonstrated their anticarcinogenic (Barrajõn‐Catalán et al. 2011; Mori et al. 2016), antinociceptive (Tahiri et al. 2017), antifungal, antibacterial and antioxidant activities (Barrajõn‐Catalán et al. 2011; Lukas et al. 2021; Mastino et al. 2021). It has been reported that the aerial part of *C. albidus* contains phenolic acids (cynarin, caftaric acid, caffeic acid, gallic acid, quinic acid, 3‐caffeoylquinic acid, 3‐p‐coumaroylquinic acid) (Barrajõn‐Catalán et al. 2011; Gonçalves et al. 2013; Lukas et al. 2021; Mastino et al. 2021; Qa'dan et al. 2003; Tomás‐Menor et al. 2013) and flavonoids (−)‐(Epi)catechin, (−)‐(Epi) gallocatechin, Glucogallin, Luteolin‐7‐ O ‐rutinoside, myricetin glycoside, kaempferol 3‐O‐rutinoside, myricitrin, prunin, rutin, quercitrin, quercetin 3‐ O‐ (2′‐caffeoyl)‐rutinoside, quercetin 3‐ O ‐glucoside, quercetin 3,4‐diglucoside, quercetin 3‐ O ‐(2′‐cumaroyl)‐ rutinoside, quercetin, quercetin glucoside (Barrajõn‐Catalán et al. 2011; Lukas et al. 2021; Mastino et al. 2021; Qa'dan et al. 2003; Tomás‐Menor et al. 2013).

Therefore, in these investigations, we evaluated the in vitro anti‐hyperglycemic properties of *C. albidus* via *α*‐amylase inhibitory activity and attempted to investigate, for the first time, the in vivo antidiabetic activity. In addition, we chemically characterized our aqueous extract by HPLC to identify the bioactive components responsible for this effect. Simultaneously, we have incorporated the *in silico* docking method to investigate various developed interactions of ligand with its target receptor proteins (Naseer et al. 2026).

## 2 Material and Methods

### 2.1 Chemical Reagents and Standard Compounds

Standard compounds: vanillic acid, gallic acid, hydroxybenzoic acid, rutin, caffeic acid, *p*‐coumaric acid, quercetin, and the pancreatic *α*‐amylase enzyme were obtained from Sigma Aldrich (USA). Alloxan and anhydrous D (+) glucose were obtained from Merck KGaA (Germany). The commercial drug acarbose is purchased from Pharma5 (Morocco).

### 2.2 Plant Material

The *C. albidus* plants were collected in March 2022 from the Taounate region in Morocco (N: 34°35′2, W: 4°38′38, altitude: 488 m). A voucher specimen with the number “0012023KC1” was deposited in the Herbarium of the Department of Biology, Faculty of Sciences, Meknes, Morocco (Mohan et al. 2026).

### 2.3 Preparation of Plant Extracts

100 g of leaves of *C. albidus* were macerated with 1000 mL of distilled water under stirring at 400 rpm for 24 h at room temperature. The solution was obtained by filtration on Whatman paper No. 1 and then evaporated under reduced pressure using a rotary evaporator at 45°C. The prepared aqueous extract was conserved at −20°C until use.

### 2.4 Animals

Wistar rats weighing 180–210 g were used in this study. These animals were bred and maintained in the animal facility at NAMAP, Taounate, Morocco. They were housed under typical environmental conditions and provided with unrestricted access to water. The experimental protocols adhered to the ethical guidelines outlined in the “Guide for the Care and Use of Laboratory Animals,” as stipulated by the National Academy of Sciences (National‐Academies‐Press 2011).

The rat was distributed into 4 groups of 6 rats each. Treatment was performed daily for 28 days.

- Normal control rats administered a daily dose of distilled water (10 mL/kg body weight).
- Diabetic control rats received daily doses of distilled water (10 mL/kg body weight).
- Diabetic rats were treated with the standard drug glibenclamide (2 mg/kg body weight).
- Diabetic rats were subjected to *Cistus albidus* aqueous extract (CAAE) at a daily dose of 500 mg/kg body weight.

### 2.5 Acute Toxicity

The acute toxicity test was carried out in accordance with guideline 423 of the Organization for Economic Co‐operation and Development (OECD) (OECD 2001). The rats were subjected to oral administration of CAAE at dosages of 500 and 2000 mg/kg body weight. Subsequently, all the animals were subjected to rigorous monitoring for 24 h. Toxicity symptoms, such as convulsions, tremors, respiratory difficulties, piloerection, and hypoactivity, were assessed after the administration of the different extract doses.

### 2.6 Phytochemical Analysis With High‐Performance Liquid Chromatography

CAAE was subjected to analysis via high‐performance liquid chromatography (HPLC) using a Jasco LC‐Net II/ADC instrument (Tokyo, Japan) equipped with a Rheodyne injector, PU‐2089 pump, and UV‐2070 UV–VIS detector, following the methodology outlined by Stoenescu et al. (2022). Phenolic compounds were detected via UV absorption at *λ* = 280 nm. Each compound was identified by its retention time and validated by comparison with standards under identical conditions (Mehmood et al. 2025).

### 2.7 Evaluation of Antidiabetic Activity In Vivo

Diabetes was experimentally induced by the intraperitoneal injection of alloxan monohydrate (150 mg/kg), following the protocol outlined by Kameswara Rao et al. (2001); Kameswara Rao and Appa Rao (2001). After 2 weeks, animals displaying blood glucose levels exceeding 200 mg/mL were chosen for the study. The assessment of the antidiabetic effects of the aqueous extract from our plant was conducted in comparison to control groups. This evaluation involved the measurement of fasting blood glucose levels and changes in body weight over days.

#### 2.7.1 Oral Glucose Tolerance Test (OGTT)

The oral glucose tolerance test was executed on rats following a 28‐day administration of the CAAE, in accordance with the procedure detailed by El Kabbaoui et al. (2016). On the final day of the experiment, rats were fasted and then orally provided with glucose (2 g/kg body weight). This glucose challenge was administered 1 h following the last dosage of the extract, the standard drug (glibenclamide), or distilled water. Blood samples (mg/mL) were obtained from the tail vein at 0, 30, 60, 90, and 120 min post‐glucose administration.

#### 2.7.2 Biochemical Analysis

Upon concluding the 28‐day experiment, the animals were sacrificed, and blood samples were obtained from all four groups. These blood samples were collected in heparin‐containing tubes. Plasma was subsequently isolated for the analysis of various parameters, including urea, ALAT, ASAT, creatinine, triglycerides, LDL‐C, HDL‐C, and total cholesterol. The quantification of these biochemical parameters was carried out using an auto‐analyzer (Architect C8000, Abbott, USA).

### 2.8 *In Vitro* Inhibition of Pancreatic *α*‐Amylase

The evaluation of antidiabetic potential was carried out in accordance with the method described by Kusano et al. (Kusano et al. 2011). Acarbose served as the reference drug, and the inhibitory effect on pancreatic α‐amylase activity was measured to determine the extent of enzyme suppression.

### 2.9 Glide Molecular Docking Methodology

Phytochemical analysis of the aqueous extract from *C. albidus* leaves revealed the presence of seven bioactive compounds, including gallic acid, hydroxybenzoic acid, vanillic acid, caffeic acid, *p*‐coumaric acid, rutin, and quercetin. These identified molecules were selected as ligands for molecular docking studies using the Maestro Glide docking software. Docking was performed against two key target proteins: one associated with antidiabetic activity (PDB ID: 1H5U) and the other with *α*‐amylase inhibition (PDB ID: 4 W93), alongside their respective co‐crystallized reference ligands.

#### 2.9.1 Protein Preparation

Seven bioactive compounds identified in the aqueous extract of *C. albidus* leaves (gallic acid, hydroxybenzoic acid, vanillic acid, caffeic acid, *p*‐coumaric acid, rutin, and quercetin) were selected as ligands for molecular docking using Maestro Glide.

The crystal structures of the antidiabetic target protein (human pancreatic *α*‐amylase, PDB ID: 1H5U) and the α‐amylase inhibitor (PDB ID: 4 W93) were obtained from the Protein Data Bank (https://www.rcsb.org) (Ali et al. 2023; Rose et al. 2011). Protein 1H5U was selected due to its well‐established role in carbohydrate digestion and glucose regulation, making it a relevant target for evaluating antidiabetic compounds. Target selection was validated based on its biological relevance, availability of high‐resolution crystal structures, presence of co‐crystallized ligands, and prior use in in silico antidiabetic studies.

Using the Protein Preparation Wizard in Glide (Maestro version 12.8) (Friesner et al. 2006), bond orders were assigned, hydrogen atoms were added, and disulfide bonds were formed. Water molecules located more than 5.0 Å from any heteroatom were removed. The protein structures were then subjected to energy minimization and optimization using the OPLS4 force field. Subsequently, a receptor grid was generated to define the ligand binding site.

Docking simulations were performed to evaluate binding affinities and interactions, providing insight into the potential antidiabetic activity of the extract's phytochemicals.

#### 2.9.2 Ligand Preparation

Seven phytochemically active compounds identified in the aqueous extract of *C. albidus* leaves were obtained from the PubChem database (https://www.ncbi.nlm.nih.gov/) and processed using the LigPrep module. These structures were converted into PDB format and optimized for molecular docking using Maestro 12.8. Low‐energy 3D conformations were generated with appropriate stereochemistry, and possible ionization states at physiological pH (7.2 ± 0.2) were assigned to each ligand to ensure accurate docking simulations.

#### 2.9.3 Receptor Grid Generation (RGG)

To enable ligand docking, the receptor grid generation (RGG) tool in Schrödinger Maestro 12.8 was employed to define the active site's location and dimensions within the protein structure. The scoring grid was generated based on the position of the co‐crystallized ligand. For nonpolar atoms in the receptor, the van der Waals (vdW) radius scaling factor was set to 1.0, and a partial charge cut‐off of 0.25 was applied to refine the docking environment.

#### 2.9.4 Protein‐Ligand Docking

Molecular docking was performed using the Glide module within Schrödinger Maestro 12.8 (Friesner et al. 2006), utilizing the previously generated receptor grid file. Docking was conducted in Standard Precision (SP) mode, with ligand flexibility enabled during sampling. To optimize ligand‐receptor interactions, the van der Waals (vdW) radius scaling factor for nonpolar ligand atoms was set to 0.80, and a partial charge cut‐off of 0.15 was applied. The vdW scaling factor was used to soften the potential, allowing minor conformational adjustments and reducing steric clashes, which improve the accuracy of predicted binding poses and interaction energies. This approach is widely recommended in Glide docking protocols to enhance the identification of energetically favorable ligand conformations. Docking results, including binding affinities and interaction patterns, were analyzed to evaluate the potential of the selected phytochemicals to interact with the antidiabetic and *α*‐amylase target proteins.

### 2.10 Statistical Analysis

All data were presented as mean values accompanied by standard deviation (mean ± SD). Statistical evaluations were performed using GraphPad Prism version 8.02. ANOVA was employed to compare CAAE, followed by Tukey's test for conducting multiple comparisons. Significance in statistics was defined as *p* ≤ 0.05.

## 3 Results and Discussion

### 3.1 Acute Toxicity

No mortality or signs of toxicity were observed within 24 h following oral administration of the aqueous extract of *C. albidus* leaves (CAAE) at doses of 500 and 2000 mg/kg body weight. The 24‐h observation period is a standard parameter for acute toxicity assessment, as recommended by OECD Guideline 423 (OECD 2001) and reported in numerous studies (Lorke 1983). Furthermore, the animals were monitored for a total of 14 days to evaluate any delayed toxic effects. No mortality or adverse behavioral changes were observed during this period, confirming the safety of CAAE in the acute toxicity test.

### 3.2 Chemical Composition

**FIGURE 1:**
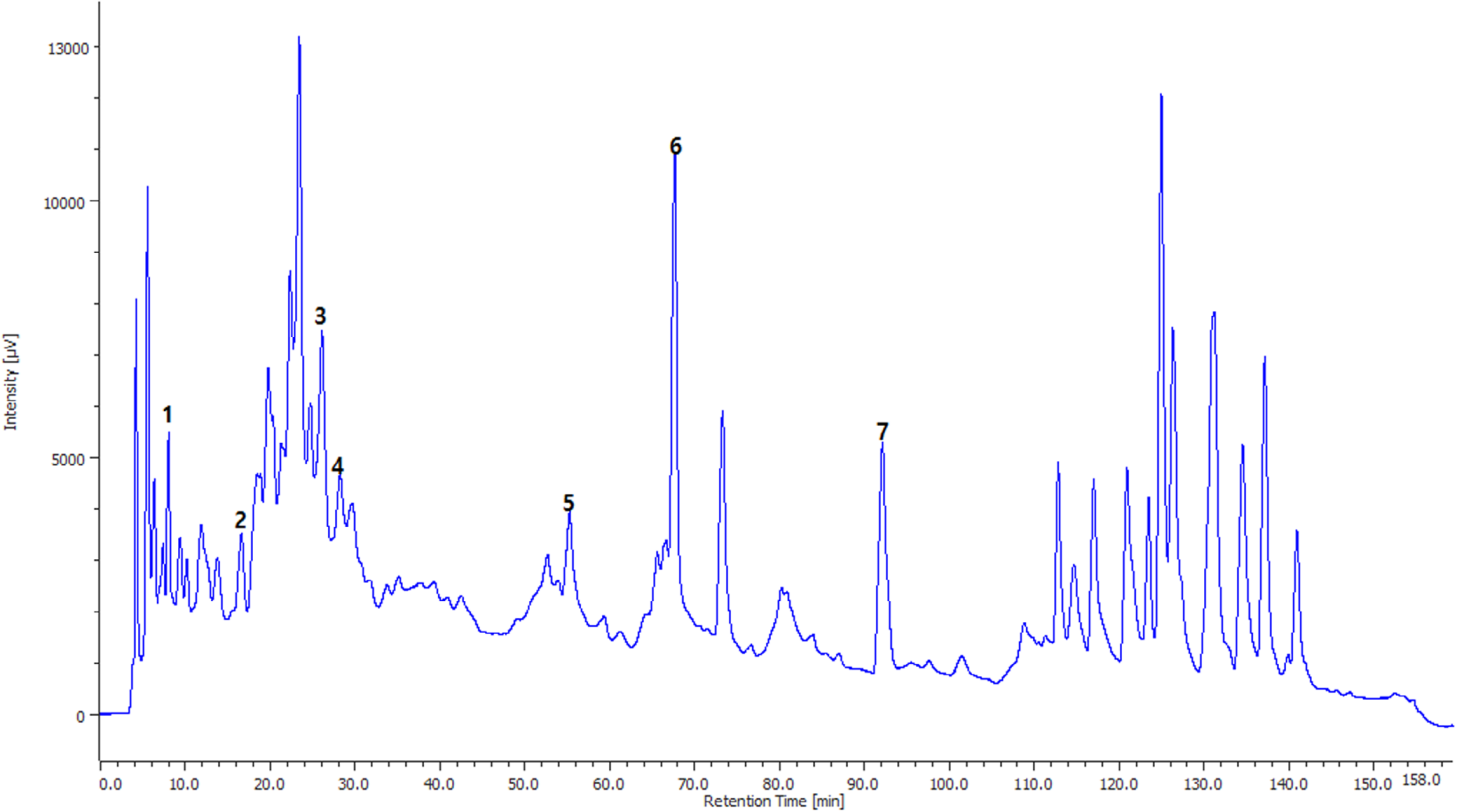
HPLC chromatogram of the aqueous extract of *C. albidus* leaves (CAAE), showing the separation of major phenolic compounds detected at the selected wavelength.

**FIGURE 2:**
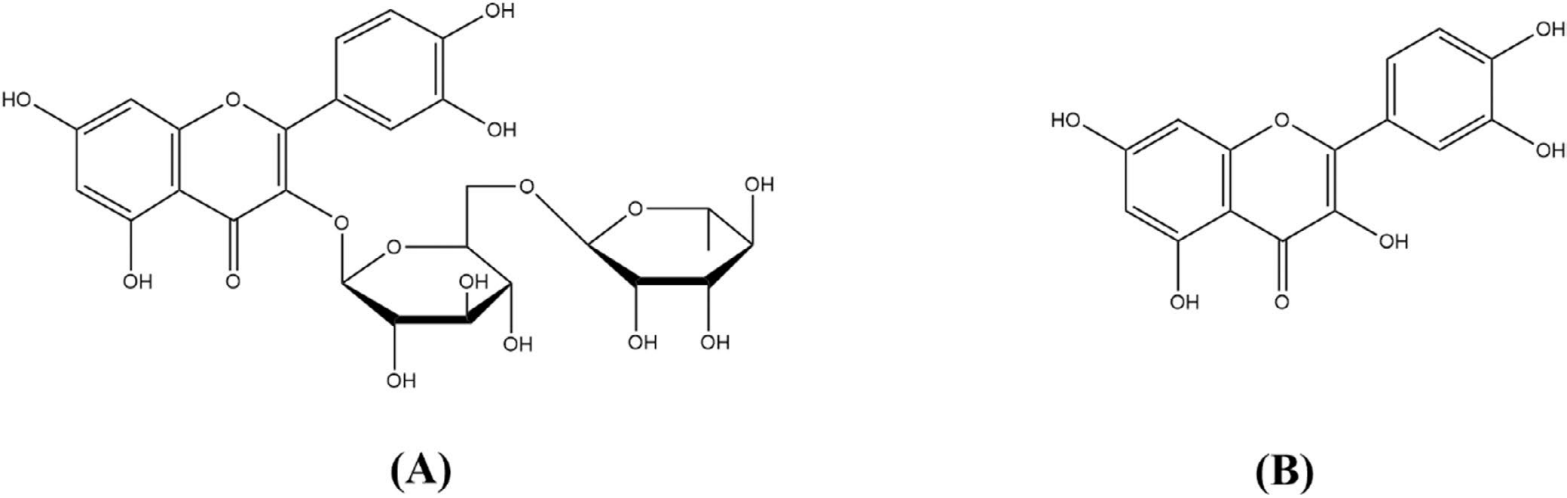
The structures of rutin (A) and quercetin (B).

The extraction yield of the aqueous extract (CAAE) was 11.2%. The chemical constituents present in the CAAE leaves were analyzed using HPLC by comparing their retention times with those of standard compounds. The HPLC chromatogram displaying the identified polyphenols is illustrated in Figure 1. Additionally, Figure 2 provides the chemical structures of the major compounds detected in the aqueous extract (CAAE). Among the phenolic compounds, rutin was found to be the most abundant, comprising 7.07% of the extract, followed by quercetin at 5.88%. Several other compounds were also identified in CAAE, but they were present in lower percentages, including vanillic acid (3.26%), p‐coumaric acid (1.91%), gallic acid (1.44%), hydroxybenzoic acid (1.33%), and caffeic acid (0.97%) (Table 1).

**TABLE 1:** Qualitative identification of major phenolic compounds in the aqueous extract of *C. albidus* leaves (CAAE) by HPLC.

Peak number	Retention time (min)	Compounds	PubChem number	Molecular formula	Area %
1	8.017	Gallic acid	370	C_7_H_6_O_5_	1.448
2	16.575	Hydroxybenzoic acid	135	C_7_H_6_O3	1.330
3	26.058	Vanillic acid	8468	C_8_H_8_O_4_	3.266
4	28.192	Caffeic Acid	689,043	C_9_H_8_O_4_	0.978
5	55.183	*P*‐Coumaric acid	637,542	C_9_H_8_O_3_	1.912
6	67.592	Rutin	5,280,805	C_27_H_30_O_16_	7.079
7	92.017	Quercetin	5,280,343	C_15_H_10_O_7_	5.881

In a study by Mastino et al. (Mastino et al. 2021), findings parallel to ours were reported, indicating the presence of 31 polyphenolic compounds in *C. albidus* leaves, encompassing various phenolic classes such as phenolic acids, glycosides, flavanols, isoflavones, anthocyanins, and caffeic acid esters. Similarly, in our previous investigation (Zouhri et al. 2024), a slightly higher number of phenolic compounds (34) was identified, with catechin, quercetin, and gallic acid as the major constituents, using UPLC‐MS/MS analysis. The difference in the number of detected compounds may be attributed to variations in analytical sensitivity, extraction procedures, and plant‐related factors such as geographical origin and environmental conditions. In particular, the high sensitivity and resolution of UPLC‐MS/MS likely enabled the detection of additional minor phenolic compounds. Furthermore, several studies have reported the presence of gallic acid (Barrajõn‐Catalán et al. 2011; Gonçalves et al. 2013), caffeic acid (Lukas et al. 2021), rutin (Tomás‐Menor et al. 2013), and catechin (Qa'dan et al. 2003) in *C. albidus* , supporting the consistency of our findings with the existing literature.

### 3.3 *In Vitro* Inhibition of Pancreatic *α*‐Amylase

**FIGURE 3:**
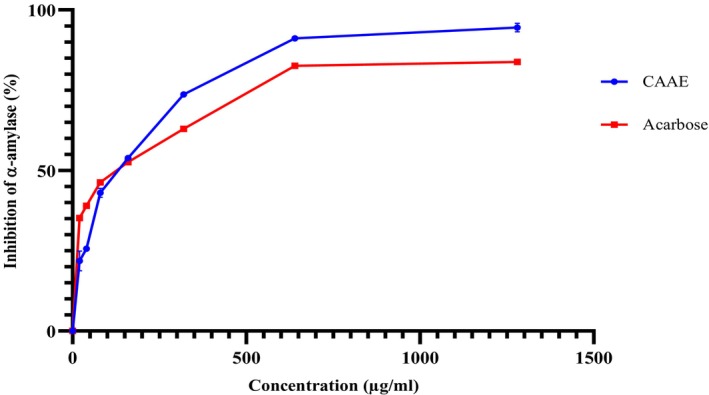
In vitro inhibition of pancreatic *α*‐amylase by the aqueous extract of *C. albidus* leaves (CAAE), expressed as percentage of inhibition at different concentrations. Values are presented as mean ± SD (*n* = 3).

**FIGURE 4:**
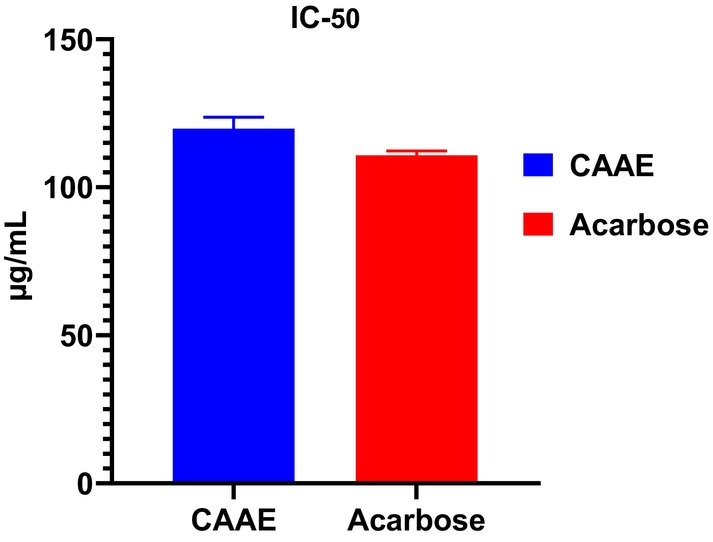
IC_50_ values of the aqueous extract of *C. albidus* leaves (CAAE) and acarbose against pancreatic *α*‐amylase, indicating the concentration required to inhibit 50% of enzyme activity. Values are expressed as mean ± SD (*n* = 3).

In this research, we studied the in vitro inhibitory activity of CAAE on pancreatic *α*‐amylase. The tested activity demonstrated a dose‐dependent response, as shown in Figure 3, and the results were expressed as IC_50_ values, which are presented in Figure 4. At a 1280 μg/mL concentration, CAAE exhibited a maximum inhibition of *α*‐amylase activity at 94.51%, while acarbose showed an 83.94% inhibition. The IC_50_ values for CAAE and acarbose were 119.81 ± 3.82 and 110.80 ± 1.44 μg/mL, respectively.

The IC_50_ value represents the concentration required to inhibit 50% of the enzyme activity; thus, a lower IC_50_ indicates a more potent inhibitor. In this study, the slightly higher IC_50_ of CAAE compared to acarbose suggests that acarbose is marginally more potent at intermediate concentrations. However, CAAE achieved a higher maximum inhibition than acarbose at the highest tested dose, indicating that while its potency at lower concentrations is slightly lower, it can reach comparable or greater inhibitory effects at higher concentrations.

Indeed, Sayah et al. (Sayah et al. 2017) demonstrated that the aqueous extracts of *C. salviifolius* and *C. monspeliensis* showed inhibitory effects on pancreatic α‐amylase, with IC_50_ values of 217.10 and 886.10 μg/mL, respectively. Notably, these IC_50_ values are higher than those observed with our extract. This disparity can be attributed to the presence of phenolic compounds, well‐recognized for their capacity to impede carbohydrate‐hydrolyzing enzyme activities (Shobana et al. 2009), with caffeic acid being a prominent example (Oboh et al. 2015). In the work of Taslimi et al. (Taslimi and Gulçin 2017), certain phenolic compounds such as *p*‐coumaric acid, resveratrol and rosmarinic acid were shown to inhibit α‐amylase in vitro, with IC_50_ values of 737.23, 376.22, and 137.36 nM respectively.

### 3.4 *In Vivo* Antidiabetic

#### 3.4.1 Changes in Body Weight

**FIGURE 5:**
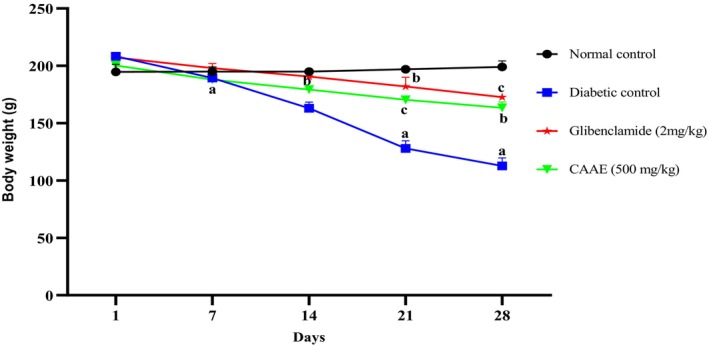
Impact of daily oral administration of CAAE on body weight in rats with alloxan‐induced diabetes. Results are presented as mean ± SD (*n* = 6). (a) *p* < 0.01 compared to the normal control group; (b) *p* < 0.05, (c) *p* < 0.01 compared to the diabetic control group.

Figure 5 presents the effects of CAAE on body weight in the study subjects. Over the course of the experiment, the diabetic control group exhibited a significant decline in body weight compared to the normal group (*p* < 0.01). However, diabetic rats treated with either CAAE or glibenclamide showed similar trends in body weight changes during the study period. Notably, by day 28, body weights in the groups receiving the plant extract and glibenclamide were significantly higher than those in the diabetic control group (*p* < 0.05 and *p* < 0.01, respectively), as depicted in Figure 5.

Weight loss is a well‐documented symptom of alloxan‐induced diabetes, which our findings also confirmed. Both CAAE and glibenclamide treatments effectively countered this weight reduction in diabetic rats compared to untreated diabetic controls. The substantial weight loss seen in the diabetic control animals is largely attributed to insulin deficiency, which triggers the breakdown of structural proteins and lipids, leading to reduced body mass (Stephen Irudayaraj et al. 2012). Insulin's essential role in glucose regulation helps prevent the catabolism of vital proteins and fats for energy, thereby preserving body weight. Treatment with CAAE and glibenclamide appeared to improve glucose metabolism, stabilize blood sugar levels, and ultimately contribute to the restoration of body weight in diabetic rats.

#### 3.4.2 Hypoglycemic Effect of CAAE

**FIGURE 6:**
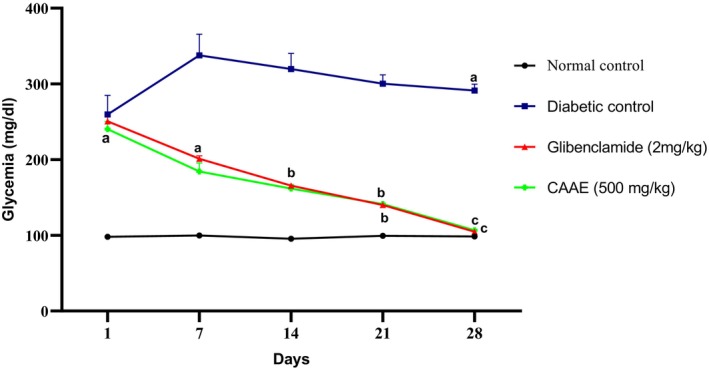
Influence of daily oral treatment with CAAE on blood glucose levels in rats rendered diabetic by alloxan. Data are shown as mean ± SD (*n* = 6). (a) *p* < 0.01 versus normal control; (b) *p* < 0.05, (c) *p* < 0.01 versus diabetic control.

Figure 6 illustrates the effects of CAAE leaf extract and glibenclamide on blood glucose concentrations in alloxan‐induced diabetic rats. Daily oral administration of CAAE resulted in a significant decrease in fasting blood glucose levels as early as day 7 when compared to the normal control group (*p* < 0.05). By the end of the 28‐day treatment period, CAAE produced a 36.61% reduction in blood glucose relative to the untreated diabetic group.

The effects of CAAE on blood glucose levels in rats resemble those of glibenclamide, potentially inducing hypoglycemia by promoting insulin release and improving its effectiveness. This can result in enhanced cellular glucose uptake and utilization. Within the plant's extracts, there may exist bioactive compounds that possess the ability to sensitize insulin receptors, making cells more responsive to insulin, or stimulate pancreatic islet β‐cells to secrete insulin. In the streptozotocin‐induced type 1 diabetic rat model, oral administration of 100 mg/kg rutin significantly decreased fasting blood glucose levels by promoting insulin secretion (Al‐Enazi 2014). In addition, further research by Gaballah et al. (2017), demonstrated that quercetin was able to increase insulin sensitivity using a type 2 diabetic rat model induced by a diet rich in fat and streptozotocin. These mechanisms could contribute to an overall enhancement of carbohydrate‐metabolizing enzymes, effectively re‐establishing normal blood glucose levels.

#### 3.4.3 OGTT of CAAE

**FIGURE 7:**
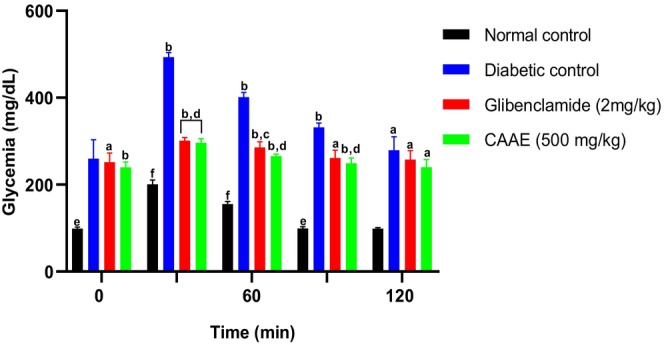
Impact of daily oral CAAE treatment on blood glucose levels during the oral glucose tolerance test (OGTT) in alloxan‐induced diabetic rats. Data are presented as mean ± SD (*n* = 6). (a) *p* < 0.05, (b) *p* < 0.01 versus normal control group; (c) *p* < 0.05, (d) *p* < 0.01 versus diabetic control group; (e) *p* < 0.05, (f) *p* < 0.01 versus Glibenclamide (2 mg/kg) group.

OGTT of CAAE leaves in normal and alloxan‐induced diabetic rats is illustrated in Figure 7. As shown in Figure 7, the oral glucose challenge induced a maximal increase in fasting blood glucose in both diabetic and normal control groups 30 min after glucose administration.

Pretreatment with CAAE (500 mg/kg) resulted in a significant reduction in blood glucose levels (252.33 ± 7.02 mg/dL; *p* < 0.01) compared to the diabetic control group (328.33 ± 6.42 mg/dL) at the 90‐min mark. Interestingly, this reduction was greater than that observed with glibenclamide (2 mg/kg), which lowered blood glucose to 358 ± 4.58 mg/dL at the same time point. This suggests that CAAE at the tested dose may exert a stronger acute antihyperglycemic effect than glibenclamide in alloxan‐induced diabetic rats.

The observed effect of CAAE could be attributed to multiple mechanisms, including stimulation of residual insulin secretion, improvement of insulin sensitivity, enhanced glucose uptake by peripheral tissues, and regulation of carbohydrate‐metabolizing enzymes (Gupta et al. 2012).

Similar findings have been reported for other *Cistus* species. For example, El Kabbaoui et al. (El Kabbaoui et al. 2016) demonstrated that the aqueous leaf extract of *Cistus ladaniferus* (500 mg/kg) significantly reduced blood glucose levels in streptozotocin‐induced diabetic rats and improved glucose tolerance, with an effect comparable to that of glibenclamide. These observations confirm the antihyperglycemic potential of *Cistus* extracts and suggest that CAAE may act through similar mechanisms. Therefore, the present results highlight the potential of CAAE as a natural antidiabetic agent, supporting further investigation into its mechanism of action.

#### 3.4.4 The Effect on Biochemical Parameters

Table 2 presents the effects of CAAE on several biochemical parameters, including ALAT, ASAT, total cholesterol, triglycerides, LDL‐C, HDL‐C, creatinine, and urea. Compared to the normal control group, diabetic rats showed a significant elevation in total cholesterol levels (*p* < 0.01), whereas serum triglyceride levels did not increase significantly in this study. Treatment with CAAE led to a marked reduction in both total cholesterol and triglycerides relative to the diabetic control group. Interestingly, the effects of CAAE were comparable to those of glibenclamide, suggesting that the extract exerts potent antihyperglycemic and hypolipidemic activity. This similarity may be attributed to the presence of bioactive phenolic compounds in CAAE, such as catechins, quercetin, and gallic acid, which are known to improve insulin sensitivity, stimulate residual insulin secretion, and modulate key enzymes involved in carbohydrate and lipid metabolism.

**TABLE 2:** Effects of the aqueous extract of *C. albidus* leaves (CAAE) on biochemical parameters in alloxan‐induced diabetic rats, including liver enzymes (ALAT, ASAT), lipid profile (total cholesterol, triglycerides, LDL‐C, HDL‐C), and renal function markers (creatinine and urea).

Biochemical parameters	Groups			
	Normal control	Diabetic control	Glibenclamide (2 mg/kg b.w.)	CAAE (500 mg/kg b.w.)
Total cholesterol (g/L)	0.55 ± 0.11	0.86 ± 0.07^b^	0.58 ± 0.05	0.64 ± 0.03^c^
Triglycerides (g/L)	0.67 ± 0.05	0.92 ± 0.08	0.69 ± 0.04	0.70 ± 0.02
LDL‐C (g/L)	0.12 ± 0.01	0.19 ± 0.10	0.13 ± 0.02	0.15 ± 0.03
HDL‐C (g/L)	0.49 ± 0.12	0.33 ± 0.03	0.45 ± 0.05	0.44 ± 0.01
ASAT (IU/L)	97.66 ± 3.05^e^	128.66 ± 6.11^a^	113 ± 7.21^d^	118 ± 6.08^c^
ALAT (IU/L)	65.66 ± 4.16	79.66 ± 6.65	67.33 ± 5.42	69.33 ± 1.52
Creatinine (mg/L)	5.60 ± 0.17	7.40 ± 0.55^a^	6.16 ± 0.35	6.54 ± 0.39
Urea (g/L)	0.57 ± 0.13	0.94 ± 0.21	0.64 ± 0.16^c^	0.66 ± 0.19

*Note:* Values are expressed as mean ± SD, *n* = 6.

^a^ *p* < 0.05.

^b^ *p* < 0.01 versus normal control group.

^c^ *p* < 0.05.

^d^ *p* < 0.01 versus diabetic control group.

^e^ *p* < 0.05 versus Glibenclamide (2 mg/kg) group.

Beyond glucose and lipid lowering, CAAE may offer additional advantages over glibenclamide, including a natural origin, lower risk of adverse effects, antioxidant activity, protection of pancreatic β‐cells, and multifactorial metabolic benefits. LDL‐C and HDL‐C concentrations remained statistically similar across all groups (*p* > 0.05), indicating that the extract selectively improved certain lipid parameters while maintaining overall lipid homeostasis. These findings underscore the potential of CAAE as a natural antidiabetic agent with both glycemic and lipid‐modulating properties, warranting further investigation into its mechanisms of action.

Regarding ALAT levels, the findings indicated remarkable similarity between the normal control and diabetic rats treated with CAAE and glibenclamide, with values of approximately 65.66, 69.33, and 67.33 IU/L (international units per liter), respectively. In contrast, untreated diabetic rats exhibited a higher level compared to these three groups, measuring 79.66 IU/L. The ASAT level in the diabetic control rats was significantly elevated compared to the other groups, reaching 128.66 IU/L.

Furthermore, there was a noteworthy increase in creatinine and urea levels in the diabetic control rats compared to the non‐diabetic control group (*p* < 0.05). However, administering the test extract and glibenclamide showed a significant decrease in creatinine and urea levels within the treated diabetic group compared to the untreated diabetic group (*p* < 0.05).

Diabetes is well‐known for causing disruptions in lipid profiles, increasing the susceptibility to coronary heart disease (Sharma et al. 2003). Perturbations in serum triglycerides and total cholesterol levels, frequently observed in diabetic patients (Orchard 1990), play a crucial role in promoting premature atherosclerosis via the elevation of these lipid components (Betteridge 1994).

Managing total cholesterol levels is crucial in mitigating diabetic complications and ameliorating lipid metabolism in individuals with diabetes. The CAAE exhibits potential hypolipidemic properties, which can offer substantial advantages to both people with diabetes and those afflicted with diabetes‐related conditions such as atherosclerosis and hyperlipidemia. In our study, the administration of the plant extract to diabetic rats resulted in a significant reduction in total cholesterol levels (Table 2). This effect could be attributed to a decrease in the activity of cholesterol bios.

In diabetes, renal metabolic changes, including a negative nitrogen balance, increased proteolysis, and diminished protein synthesis, are evident (Tuvemo et al. 1997). The administration of the CAAE to diabetic rats led to improved plasma protein levels. Notably, diabetic rats exhibited significantly higher serum urea and creatinine levels, well‐recognized indicators of renal dysfunction (Almdal and Vilstrup 1988; Honoré et al. 2012). Our study demonstrates that the CAAE resulted in a substantial reduction in serum urea and creatinine levels in diabetic rats, indicating its potential to halt the progression of kidney damage in diabetes. This observed effect could be attributed to the extract's ability to mitigate metabolic disruptions associated with protein and nucleic acid metabolism due to hyperglycemia, which was further supported by the enhancement of glucose homeostasis in our study.

Elevated ASAT and ALAT activities have been consistently reported in previous studies as indicators of active liver injury associated with diabetes (Florence et al. 2014; Kade et al. 2009). Our study's observed increase in plasma ASAT and ALAT activities is probably attributed to hepatocellular injury resulting from diabetes. This is corroborated by a previous study documenting hepatic necrosis in diabetic rats (Arunachalam and Parimelazhagan 2013). Notably, oral administration of CAAE and glibenclamide to diabetic groups reduced the activity of these enzymes in plasma.

This outcome can be attributed to various bioactive compounds in *Cistus albidus* leaves, including gallic acid (Zouhri et al. 2024). This bioactive polyphenol is known for its therapeutic properties, including anti‐hyperglycemic and insulin‐secretagogic effects in insulin‐deficient diabetic rats (Latha and Daisy 2011) (Punithavathi et al. 2011). Additionally, it exerts a protective influence on pancreatic β‐cells, guarding them against damage (Gandhi et al. 2014).

Similarly, another study has highlighted the hypoglycemic properties of flavonoids (Romano et al. 2013) found in *C. albidus* (Zouhri et al. 2024). These compounds are associated with antidiabetic activities, as they promote glucose uptake in peripheral tissues and mitigate oxidative stress in diabetes (Eid et al. 2010; Rauter et al. 2010).

Studies have shown that rutin decreases glucose absorption in the small intestine by inhibiting α‐glucosidases and α‐amylase, key enzymes in carbohydrate digestion (Ahmed et al. 2010; Jadhav and Puchchakayala 2012; Li et al. 2009). In isolated rat pancreatic islet models, rutin was found to promote a significant increase in insulin secretion (Ahmed et al. 2010; Esmaeili et al. 2009). Furthermore, rutin has demonstrated insulin‐mimetic effects in rat solar and diaphragmatic muscles (Ahmed et al. 2010; Kappel et al. 2013), by stimulating glucose transport via activation of GLUT‐4 transporter synthesis and translocation (Kappel et al. 2013). Finally, rutin increases PPARγ expression, contributing to improved insulin resistance and promoting glucose uptake in skeletal muscle and adipose tissue (Ahmed et al. 2010).

Other species of the *Cistus* genus have also been reported to have hypoglycemic effects (El Kabbaoui et al. 2016; Sayah et al. 2021). Thus, the aqueous extract of *C. ladaniferus* (500 mg/kg body weight) showed a 34% hypoglycemic activity in streptozotocin‐induced diabetic rats at the end of the experiment (El Kabbaoui et al. 2016). These results are consistent with those reported by Sayah et al. (2021), who demonstrated the hypoglycemic effects of aqueous extracts of *C. salviifolius* and *C. monspeliensis* (500 mg/kg body weight) in diabetic rats. At the end of the fourth week of the experiment, the inhibition of hyperglycemia was 56.27% for *C. salviifolius* and 57.04% for *C. monspeliensis* .

Nonetheless, there exists a necessity for bioactivity‐driven drug discovery to isolate the lead compound responsible for the antidiabetic effects and elucidate the potential mechanism(s) of action.

## 4 Molecular Docking Results

The selection of the target protein 1H5U (Glycogen Phosphorylase B) for molecular docking was based on its essential role in the regulation of glycogen metabolism, which is directly relevant to the study of potential hypoglycemic agents. Glycogen phosphorylase B is involved in the degradation of glycogen to glucose‐1‐phosphate, a key step in glucose regulation, making it an ideal target for compounds aimed at modulating blood glucose levels.

**FIGURE 8:**
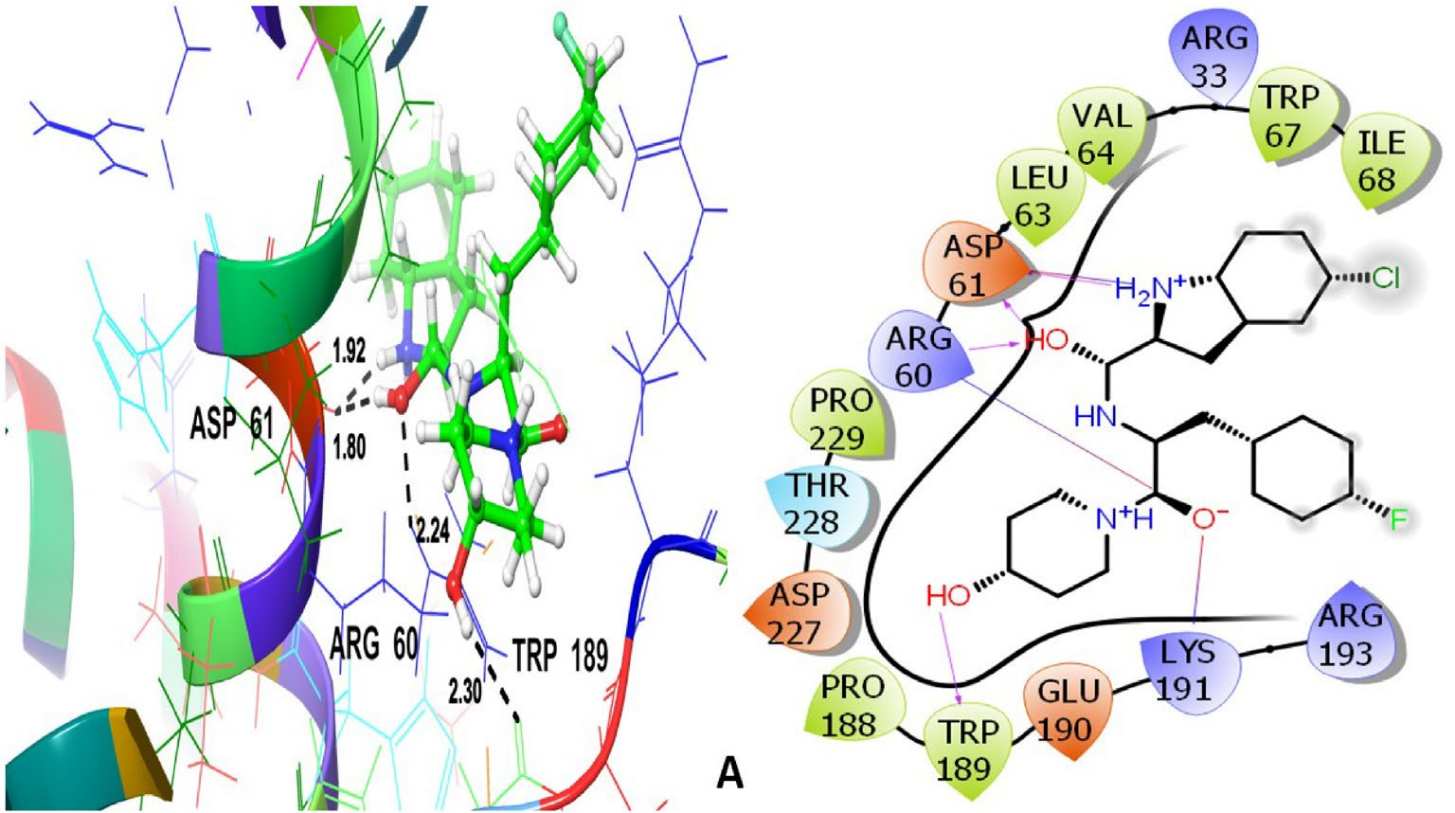
Co‐crystallized ligand (A): 3D and 2D view with an antidiabetic target protein 1H5U.

**FIGURE 9:**
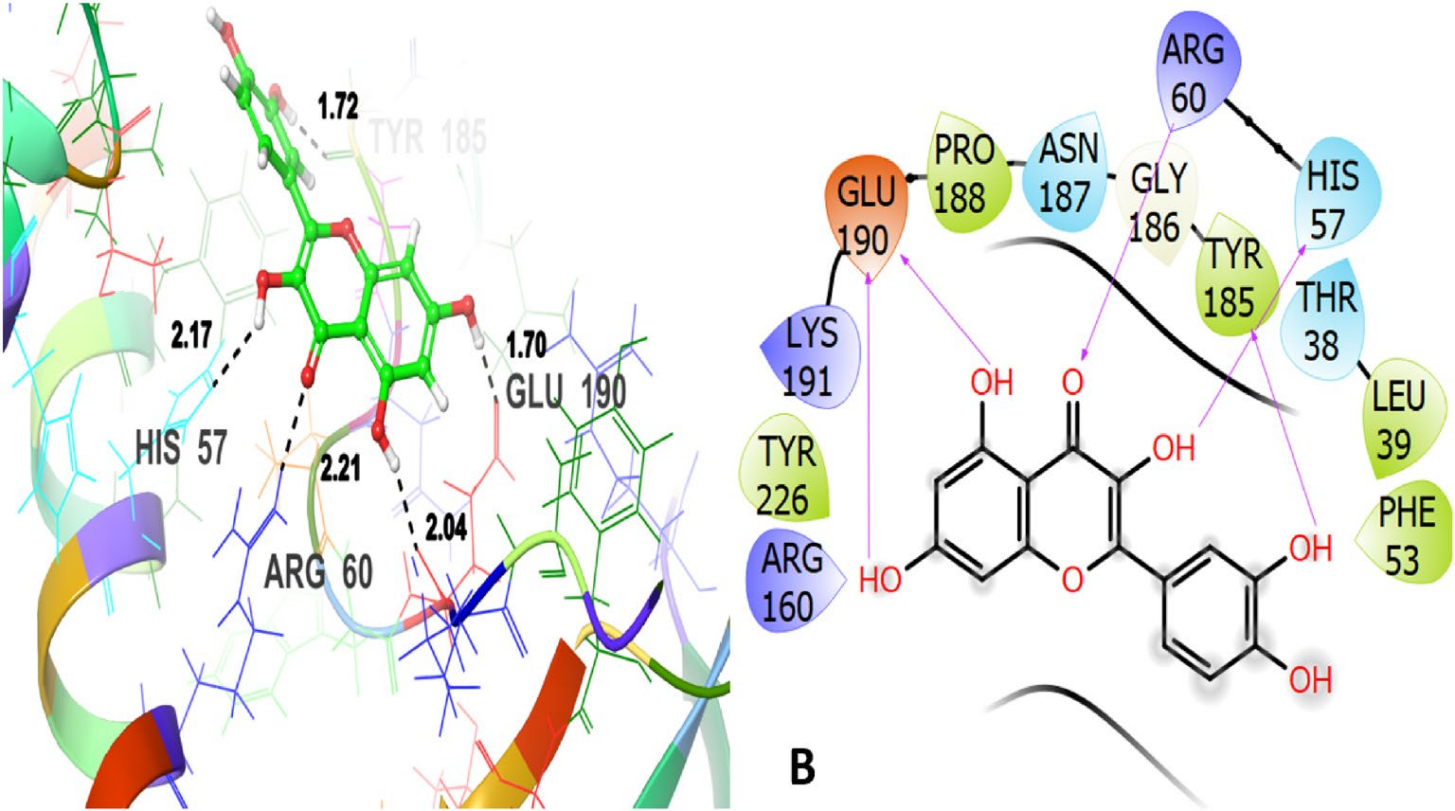
Quercetin (B)‐3D and 2D view with an antidiabetic target protein 1H5U.

Docking results of antidiabetic target protein 1H5U with different ligands of aqueous extract of *C. albidus* leaves under investigation are given in Table 3. The Co‐crystallized ligand exhibited the GScore is −5.320 kcal/mol and interacted with the target protein through hydrogen bonding with ASP61, ARG60, TRP189 at varying distances. THR228 contributed to the polar interactions. Several hydrophobic interactions were observed with LEU63, VAL64, TRP67, ILE68, PRO188, TRP189, PRO229 (Figure 8). These amino acid residues are recognized as associated with the active binding pocket and thus serve as a reference to evaluating the ligand binding consistency. These hydrophobic interactions involve nonpolar amino acids and facilitate the ligand's binding affinity. Among the tested compounds, quercetin revealed the most favorable binding affinity with the GScore is −5.523 kcal/mol and engaged in hydrogen bonding and hydrophobic interactions with the target protein (Figure 9). It formed multiple hydrogen bonding interactions with HIS57, ARG60, TYR185, GLU190. These interactions contribute to the ligand's specific positioning near the binding site, ARG60 is observed to be present in the co‐crystallized ligand. THR38, HIS57, ASN187 amino acid residues contributed to polar interactions. Hydrophobic interactions involve LEU39, PHE53, TYR185, PRO188, TYR226. These interactions are critical to stabilizing the ligand within the binding pocket. These amino acid residues coincide with those of the reference, co‐crystallized ligand, suggesting quercetin's possible antidiabetic action by occupying the same active binding area.

**TABLE 3:** Glide molecular docking data of top hit ligands of aqueous extract of *C. albidus* leaf extract against *α*‐amylase inhibitor (PDB ID: 1H5U).

Ligands interaction with (Antidiabetic) target protein 1H5U	DScore (kcal/mol)	Gscore (kcal/mol)	Glide Emodel (kcal/mol)	Polar residues	H‐bonded amino acid residues with relevant distance in Å	Hydrophobic interactions
(A) Co‐crystallized ligand	−4.902	−5.320	−38.198	THR228	ASP61 (1.80, 1.92) ARG60 (2.24) TRP189 (2.30)	LEU63 VAL64 TRP67 ILE68 TRP189 PRO188 PRO229
(B) Quercetin	−5.491	−5.523	−46.798	THR38 HIS57 ASN187	HIS57 (2.17) ARG60 (2.21) TYR185 (1.72) GLU190 (1.70)	LEU39 PHE53 TYR185 PRO188 TYR226
(C) Vanillic acid	−5.290	−5.290	−32.516	THR228	TRP189 (1.97)	LEU63 VAL64 TRP67 PRO188 TRP189 PRO229

Vanillic acid having the GScore of −5.290 kcal/mol interacted hydrophobically with LEU63, VAL64, TRP67, PRO188, TRP189, and PRO229; THR228 is the only polar amino acid residue involved. These residues also play a role in the co‐crystallized ligand's binding, indicating that vanillic acid can interact with the glycogen phosphorylase B catalytic site in an efficient manner.

**FIGURE 10:**
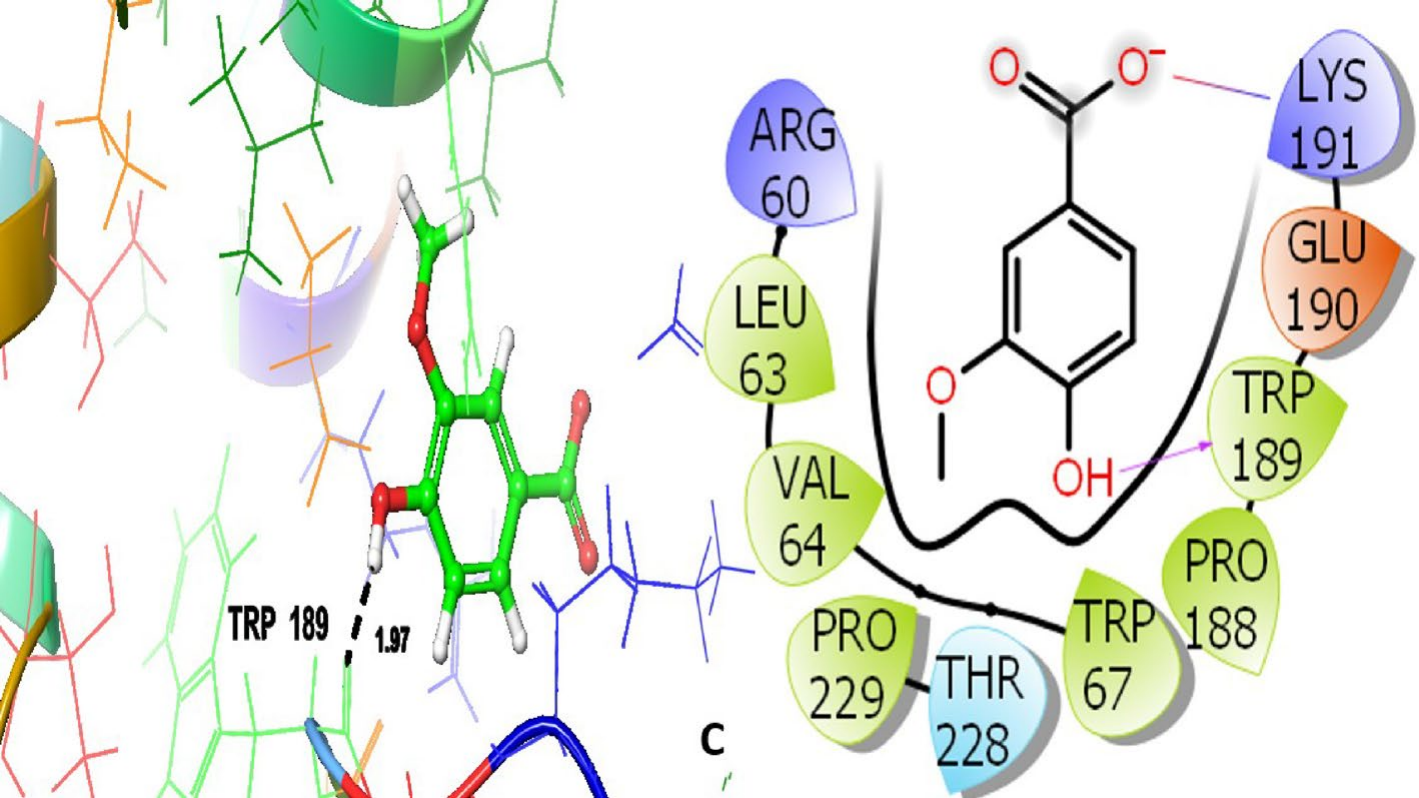
Vanillic acid (C)‐3D and 2D view with an antidiabetic target protein 1H5U.

In addition, TRP189 has established the H‐bond at 1.97 Å (Figure 10). Overall, the docking analysis indicates that rather than a single dominating ligand, the antidiabetic action of *C. albidus* extract is most likely mediated by a synergistic multi‐compound interaction with glycogen phosphorylase B.

Despite having moderate docking scores (−4.417 to −4.861 kcal/mol), other phenolic compounds such as gallic acid, caffeic acid, *p*‐coumaric acid, hydroxybenzoic acid, and rutin regularly interacted with important amino acids including ARG60, ASP61, HIS57, TRP189, and THR228 (Table S1; Figures S1–S5). The conservation of binding residues emphasizes their combined contribution to the reported in vivo hypoglycemic effect, even though their binding energies were marginally lower than quercetin's. To further explore the molecular basis of the observed in vitro α‐amylase inhibitory activity, the identified phenolic compounds were docked against human pancreatic *α*‐amylase (PDB ID: 4 W93) target protein. By regulating the digestion of carbohydrates, this enzyme is a crucial therapeutic target for lowering postprandial hyperglycemia (Maliwal et al. 2022).

**FIGURE 11:**
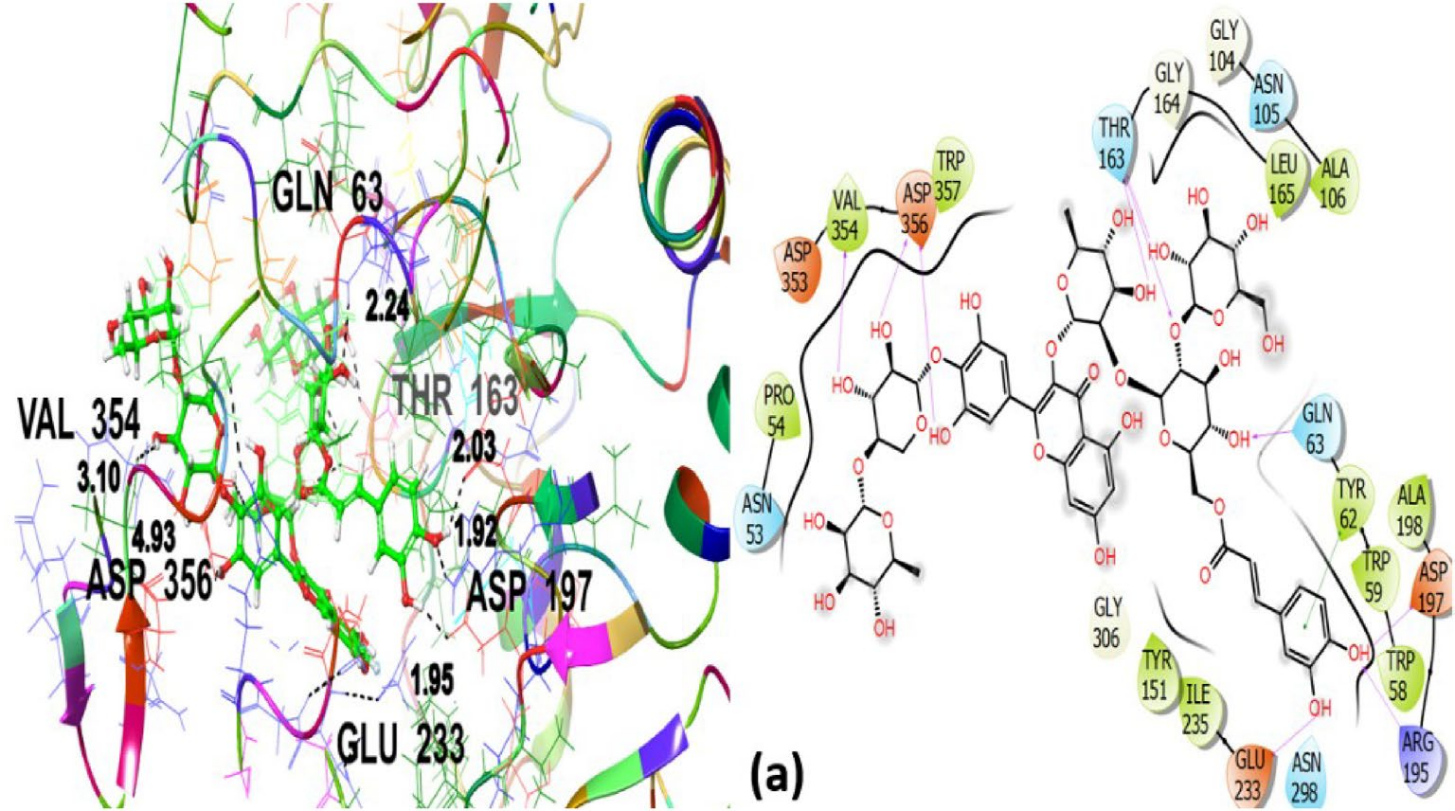
Co‐crystallized ligand (a) 3D and 2D view with α‐amylase inhibitory target protein 4 W93.

The same ligands were docked with *α*‐amylase inhibitory target protein, and outcomes with the following interpretations are presented in Table 4. Co‐crystallized ligand exhibited the strongest binding affinity with a GScore of −9.449 kcal/mol, and interacted with GLN63, THR163, ASP197, VAL354, ASP356, GLU233 amino acid residues with the target protein through hydrogen bonding. ASN53, GLN63, ASN105, THR163 and ASN298 contributed to polar interactions. A variety of hydrophobic interactions were noted with PRO54, TRP58, TRP59, TYR62, ALA106, TYR151, LEU165, ALA198, ILE235, VAL354, TRP357 (Figure 11) which involved nonpolar amino acids and thus facilitate the ligand's binding affinity. The observed residues are well‐known catalytic and substrate‐binding residues of *α*‐amylase.

**TABLE 4:** Glide molecular docking data of top hit ligands from *C. albidus* aqueous leaf extract against the human pancreatic *α*‐amylase (PDB ID: 4 W93).

Ligands interaction with (Antidiabetic) target protein 1H5U	DScore (kcal/mol)	Gscore (kcal/mol)	Glide Emodel (kcal/mol)	Polar residues	H‐bonded amino acid residues with relevant distance in Å	Hydrophobic interactions
(a) Co‐crystallized ligand	−9.473	−9.449	−141.580	ASN53 GLN63 ASN105 THR163 ASN298	GLN63 (2.24) THR163 (2.03) ASP197 (1.92) VAL354 (3.10) ASP356 (4.93) GLU233 (1.95)	PRO54 TRP58 TRP59 TYR62 ALA106 TYR151 LEU165 ALA198 ILE235 VAL354 TRP357
(b) Rutin	−7.389	−7.417	−89.491	GLN63 THR163 ASN298	TRP59 (2.73) THR163 (2.25) ARG195 (2.23) GLU233 (1.55, 2.70) ASN298 (2.73) ASP356 (1.79)	TRP59 TYR62 LEU162 LEU165 ALA198 ILE235 PHE256 TRP357
(c) Quercetin	−6.716	−6.748	−61.996	GLN63 THR163 ASN298	ARG195 (2.31) ASP197 (1.73) GLU233 (1.64)	TRP59 TYR62 LEU162 LEU165 ILE235 PHE256

**FIGURE 12:**
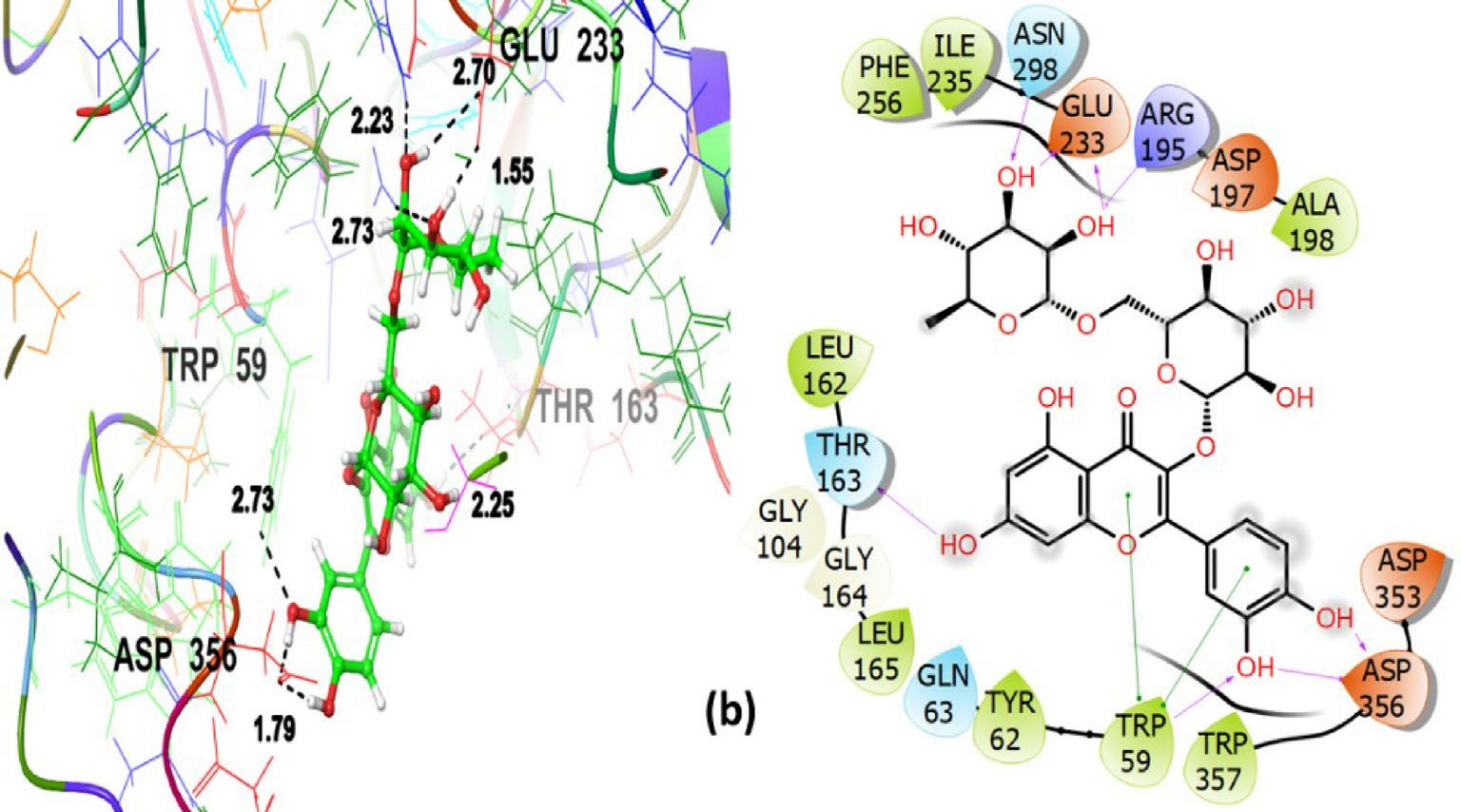
Rutin (b)‐3D and 2D view with α‐amylase inhibitory target protein 4 W93.

The rutin demonstrated the highest binding affinity (GScore −7.417 kcal/mol) and engaged in hydrogen bonding and hydrophobic interactions with the target protein amino acid residues (Figure 12). It established hydrogen bonding with TRP59, THR163, ARG195, GLU233, ASN298, ASP356 and was found to be responsible for contributing to the ligand's specific positioning near the binding site. GLN63, THR163, ASN298 amino acid residues contributed to polar interactions. Hydrophobic interactions involved TRP59, TYR62, LEU162, LEU165, ALA198, ILE235, PHE256 and TRP357 amino acid residues further stabilized the complex. The robust interaction profile of rutin supports its experimentally observed α‐amylase inhibitory potency, because these interactions are critical to stabilizing the ligand within the binding pocket.

**FIGURE 13:**
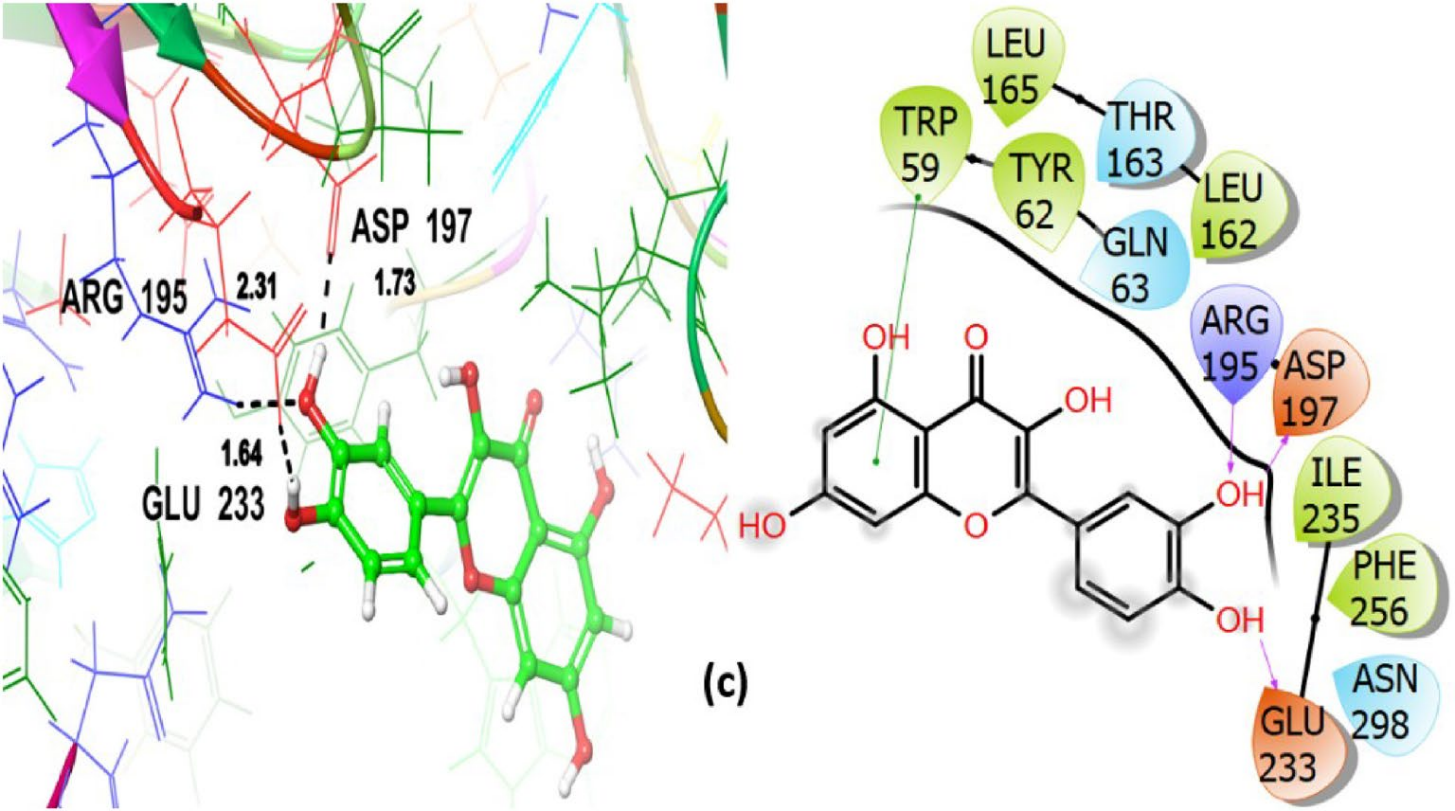
Quercetin (c)‐3D and 2D view with α‐amylase inhibitory target protein 4 W93.

Quercetin not only showed favorable binding (GScore −6.748 kcal/mol) but also interacted hydrophobically with TRP59, TYR62, LEU162, LEU165, ILE235, and PHE256. Polar amino acid residues involved are GLN63, THR163, ASN298, whereas ARG195, ASP197, GLU233 have established the H‐bond (Figure 13). In line with its well‐known enzyme inhibitory qualities, these interactions imply that quercetin binds inside the catalytic pocket and may obstruct substrate access.

Although their docking scores ranged from −4.634 to −6.152 kcal/mol, other phenolic acids such as caffeic acid, *p*‐coumaric acid, gallic acid, hydroxybenzoic acid, and vanillic acid consistently interacted with important catalytic residues like ASP197, GLN63, THR163, and ARG195 (Figures S6–S10; Table S2). Their combined binding, despite their individual weaknesses, indicates a cumulative inhibitory impact, which could account for the crude extract's high *in vitro α*‐amylase inhibition.

## 5 Conclusion

In conclusion, the single dosage of 500 mg/kg of the CAAE demonstrated robust hypoglycemic effects through inhibiting carbohydrate‐digesting enzymes and glucose absorption. It significantly mitigated hyperglycemia in alloxan‐induced diabetic rats. Notably, this botanical remedy enhanced various parameters, including the lipid profile, urinary glucose levels, body weight, and oral glucose tolerance in the diabetic rat model. Nevertheless, further investigative efforts are warranted to gain a comprehensive understanding of the precise mechanism behind this antidiabetic effect. The glide molecular docking results indicate that the GScore values of seven constituents of an aqueous extract of *C. albidus* leaves exhibited anti‐hyperglycemic and α‐amylase inhibitory activities. Consequently, the detailed analysis and visualization of docking results show the multi‐dimensional potential of *C. albidus* leaves, highlighting their capacity to modulate vital biological processes. The experimental evidence from in vitro *α*‐amylase inhibition and in vivo antidiabetic research is in good agreement with the docking results. Among the most prevalent phenolics found by HPLC analysis are compounds like quercetin and rutin, which showed excellent docking scores and stable interactions with both targets. Their function in mediating *C. albidus* antidiabetic actions is highly supported by their well‐established biological activity and advantageous binding at important catalytic residues.

Crucially, the modest docking scores for a number of phenolic acids are not indicative of weak action; rather, they support a polypharmacological process in which numerous chemicals work together to modify glucose metabolism and enzyme function. This pattern of interactions between several targets and ligands is in line with the comprehensive therapeutic benefits of plant‐based extracts. These findings not only contribute to strengthening our understanding of the isolated compounds' mechanism of action but also explore the inherent promising roles in diabetes mellitus management as well as regulation of carbohydrate metabolism.

## Author Contributions

**Aziz Zouhri, Naoual El Menyiy, Yahya El‐ mernissi, Rafik El‐mernissi:** Conceptualization, writing the original draft, reviewing and editing. **Mohamed Reda Kachmar, El Mouhri Ghita, Naima Mammate, Farhan Siddique, Rabie Kachkoul, Sumaira Nadeem:** Formal analysis, investigations, funding acquisition. **Esmael M. Alyami, and Yousef A. Bin Jardan:** Resources and project administration. **Mohammed Dauelbait, Mohammad Khalid and Lhoussain Hajji:** Data validation, data curation and supervision.

## Funding

This work is supported by the King Saud University through the Ongoing Research Funding program (ORF‐2026‐457), King Saud University, Riyadh, Saudi Arabia.

## Disclosure

*ARRIVE Guidelines*: The study was conducted with respect to ARRIVE guidelines. *Plant Collection Approval*: No approval was needed from the authority in Morocco to collect Cistus albidus (L.) for research purposes. *IUCN Policy Statement*: The collection of plant material was compiled with relevant institutional, national, and international guidelines and legislation.

## Ethics Statement

The current study was approved by the Institutional Ethical Committee of the Moulay Ismail University, Morocco (reference number 04/2019/LBEAS).

## Consent

The authors have nothing to report.

## Conflicts of Interest

The authors declare no conflicts of interest.

## Supporting information


**Data S1:** fsn371658‐sup‐0001‐supinfo.docx.

## Data Availability

Data will be available upon request from the corresponding author.
