# Supplementary material for: Antidiabetic Activity of Phenolic‐Rich Extracts From Cistus albidus (L.) in Alloxan‐Induced Diabetic Rats: In Vitro, In Vivo, and In Silico Investigations
Source: Food Sci Nutr. 2026 Apr 17;14(4):e71658. doi: 10.1002/fsn3.71658 (PMC13088270; doi:10.1002/fsn3.71658)
Supplement: Supplementary file 1 — Data S1: fsn371658‐sup‐0001‐supinfo.docx. [file FSN3-14-e71658-s001.docx]

**Antidiabetic activity of phenolic-rich extracts from *cistus albidus* (L.) in alloxan-induced diabetic rats: *in vitro*, *in vivo*, and *in silico* investigations**

Aziz Zouhri^1,2*^, Naoual El Menyiy^2^, Yahya El-mernissi^3^, Rafik El-mernissi^1^, Mohamed Reda Kachmar^4,5^, Farhan Siddique^6^, Rabie Kachkoul^7,8^, Sumaira Nadeem^9^, El Mouhri Ghita^7^, Naima Mammate^10^, Yousef A. Bin Jardan^11^, Esmael M. Alyami^12,13^, Mohammed Dauelbait^14*^, Mohammad Khalid^15^, Lhoussain Hajji^1*^

**Supplementary Docking Data**

# Table S1: Glide molecular docking data of *various* ligands of aqueous extract of *C. albidus* leaf extract against *α*-amylase inhibitor (PDB ID: 1H5U)*.*

| **Ligands interaction with (Antidiabetic) target protein 1H5U** | **DScore**  **(kcal/mol)** | **Gscore**  **(kcal/mol)** | **Glide Emodel**  **(kcal/mol)** | **Polar residues** | **H-bonded amino acid residues with relevant distance in Å** | **Hydrophobic interactions** |
| --- | --- | --- | --- | --- | --- | --- |
| D)Hyd-roxybenzoic acid | -4.861 | -4.861 | -27.281 | THR228 | TRP189  (2.14) | LEU63,  VAL64,  TRP67,  PRO188,  TRP189, PRO229 |
| E)p-coumaric acid | -4.598 | -4.598 | -29.364 | THR228 | TRP189  (2.01),  LYS191  (1.73) | LEU63,  VAL64,  TRP67,  PRO188,  TRP189, PRO229 |
| F)Gallic acid | -4.478 | -4.484 | -29.969 | HIS57 | ASP61  (1.65,1.81), ARG60  (1.95),  LYS191  (2.66) | VAL64 |
| G)Rutin | -4.438 | -4.467 | -58.904 | THR38,  HIS57,  ASN187 | ARG60  (2.18, 2.50), TYR185  (1.89), GLU190  (1.92)  ALA192  (2.66) | LEU39,  PHE53,  LEU183,  TYR185,  ALA192  TYR226 |
| H)Caffeic Acid | -4.417 | -4.417 | -35.397 | THR228 | LYS191  (1.67) | LEU63,  VAL64,  TRP67,  TRP189,  PRO229 |


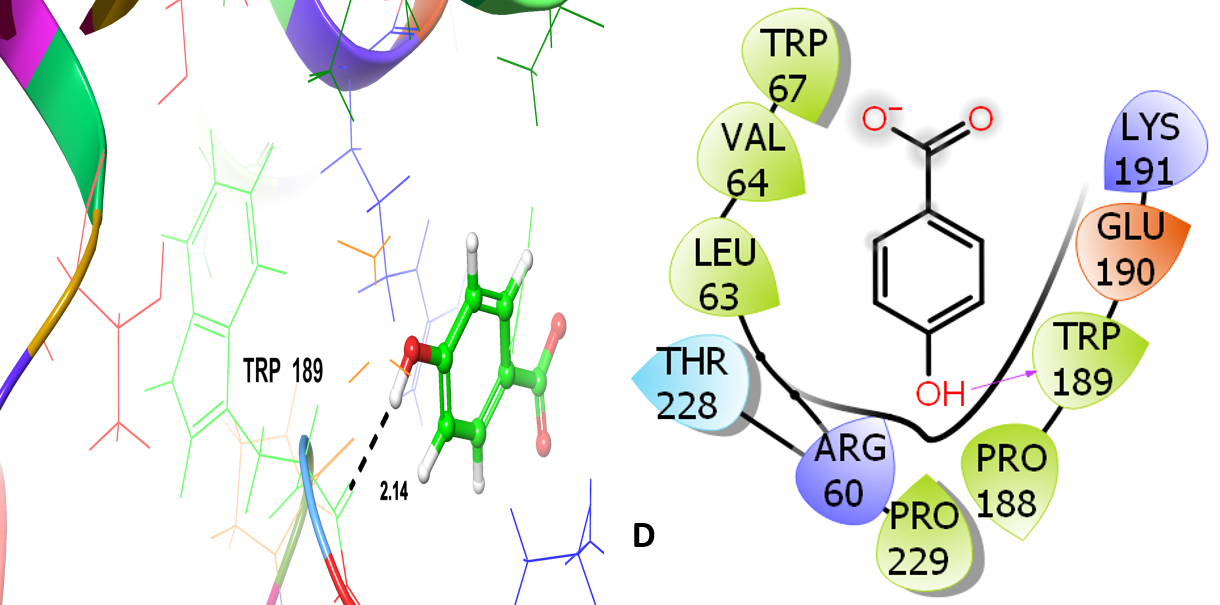


# Figure S1: Hydroxybenzoic acid (D)-3d and 2d view with an antidiabetic target protein 1H5U.


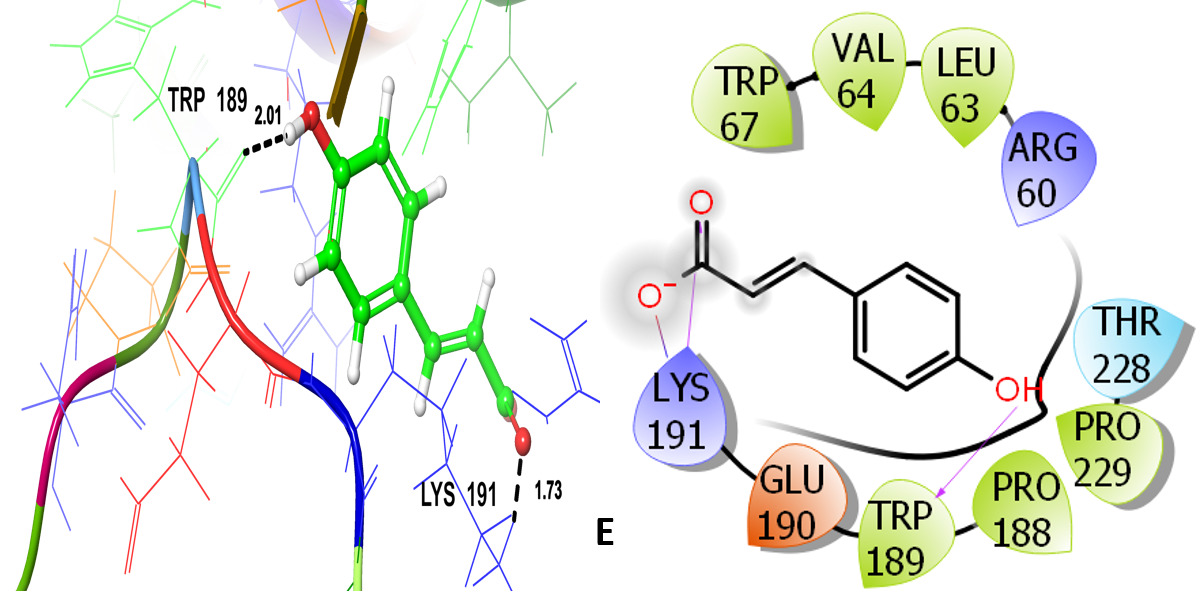


# Figure S2: *p*-Coumaric acid (E)-3d and 2d view with an antidiabetic target protein 1H5U.


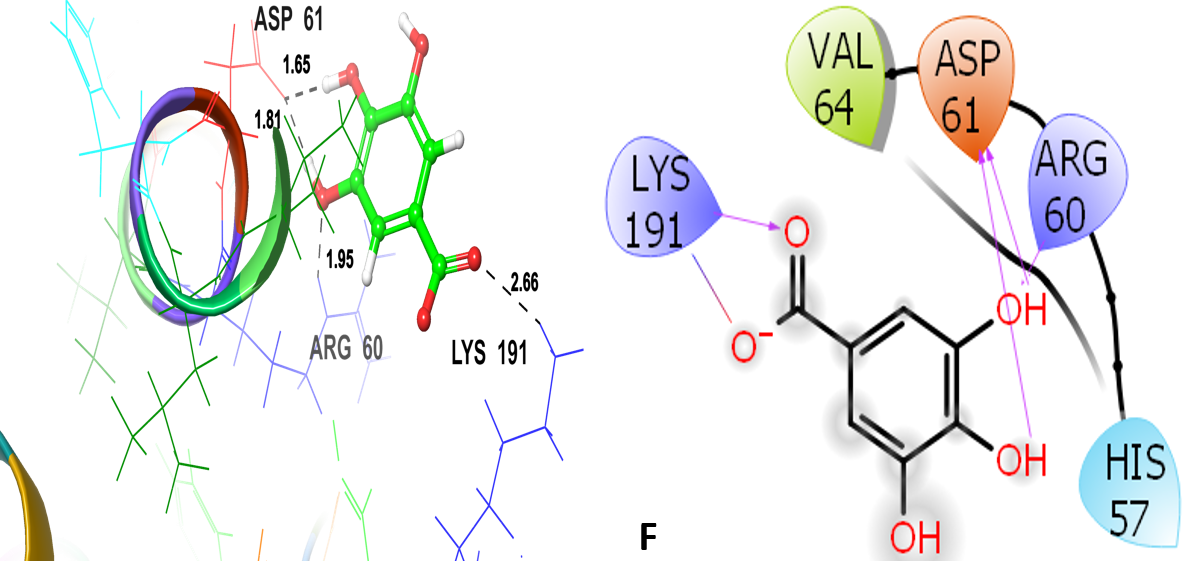


Figure S3: Gallic acid (F)-3d and 2d view with an antidiabetic target protein 1H5U.


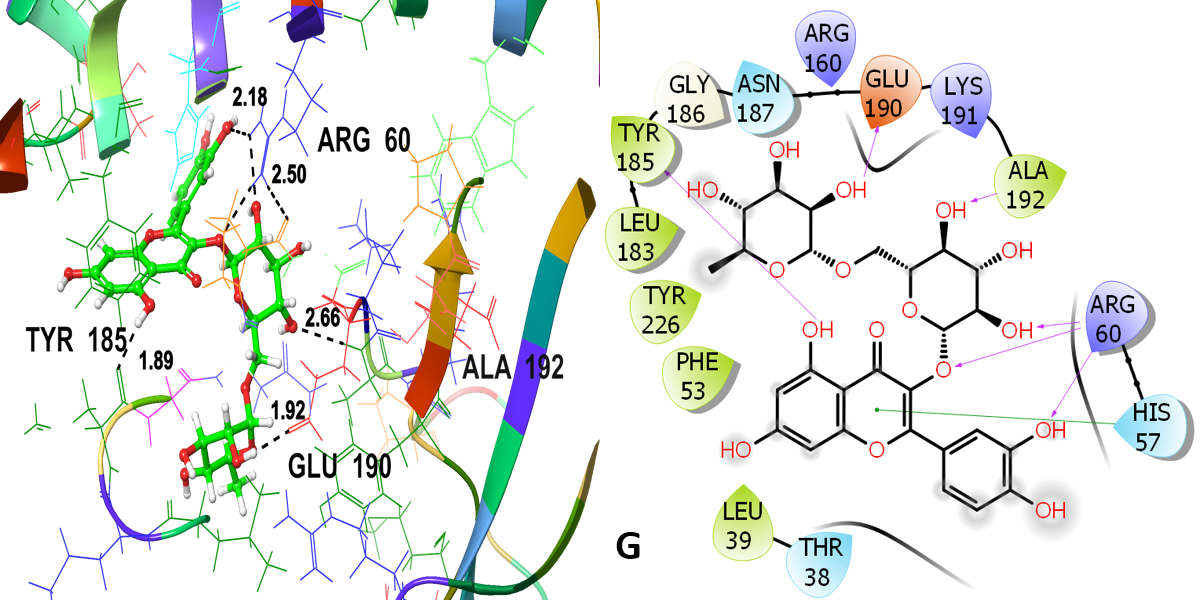


# Figure S4: Rutin (G)-3d and 2d view with an antidiabetic target protein 1H5U.


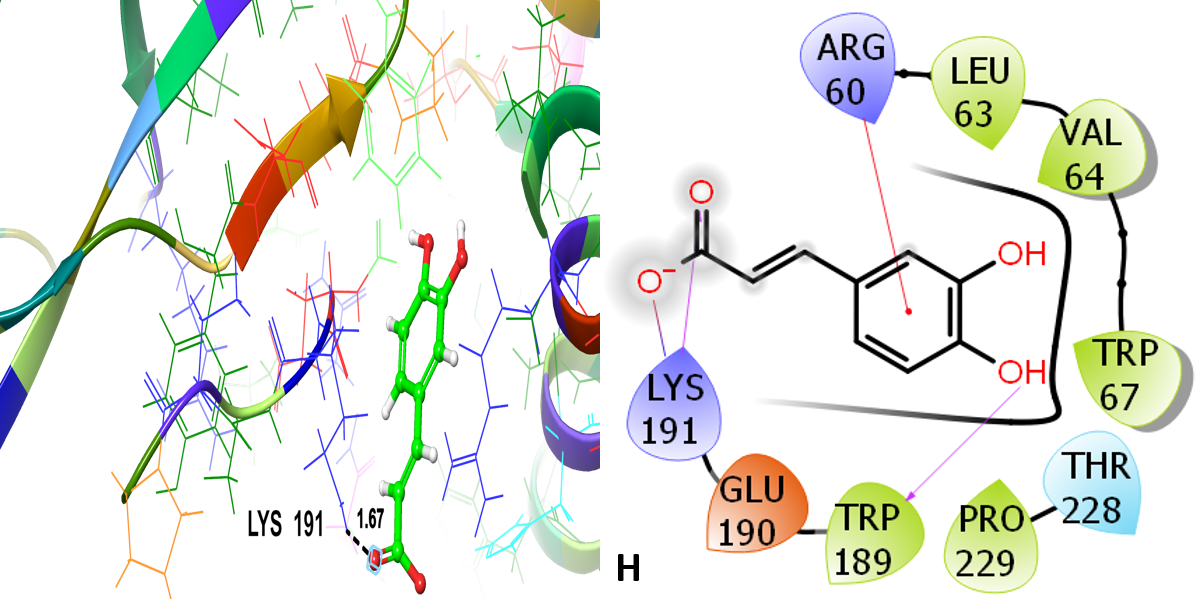


# Figure S5: Caffeic Acid (H)-3d and 2d view with an antidiabetic target protein 1H5U.

# Table S2: Glide molecular docking data of various ligands with an aqueous extract of *C. albidus* leaves against the human pancreatic *α*-amylase (PDB ID: 4W93).

| **Ligands interaction with (Antidiabetic) target protein 1H5U** | **DScore**  **(kcal/mol)** | **Gscore**  **(kcal/mol)** | **Glide Emodel**  **(kcal/mol)** | **Polar residues** | **H-bonded amino acid residues with relevant distance in Å** | **Hydrophobic interactions** |
| --- | --- | --- | --- | --- | --- | --- |
| (d) Caffeic Acid | -6.152 | -6.152 | -41.412 | GLN63, THR163, | GLN63  (1.45), ASP197  (2.12) | TRP59, TYR62,  VAL98,  LEU162,  LEU165,  ALA198, |
| (e) *p*-coumaric acid | -5.883 | -5.883 | -34.857 | GLN63, THR163, | GLN63  (1.79), ASP197  (1.48) | TRP59, TYR62,  LEU162,  LEU165,  ALA198 |
| (f) Gallic acid | -4.927 | -4.934 | -35.826 | ASN298 | ASP197  (1.81, 1.93) | TYR62,  TYR151,  LEU162,  LEU165,  ALA198,  ILE235 |
| (g) Hydroxybenzoic acid | -4.839 | -4.839 | -26.915 | ASN298 | ARG195  (2.28), ASP197  (1.74) | TRP59, TYR62,  VAL98,  LEU162,  LEU165,  ALA198 |
| (h) Vanillic acid | -4.634 | -4.634 | -30.582 | SER199 | LYS200  (2.45),  ILE235  (1.75) | TYR151,  LEU162,  ALA198,  VAL234,  ILE235 |


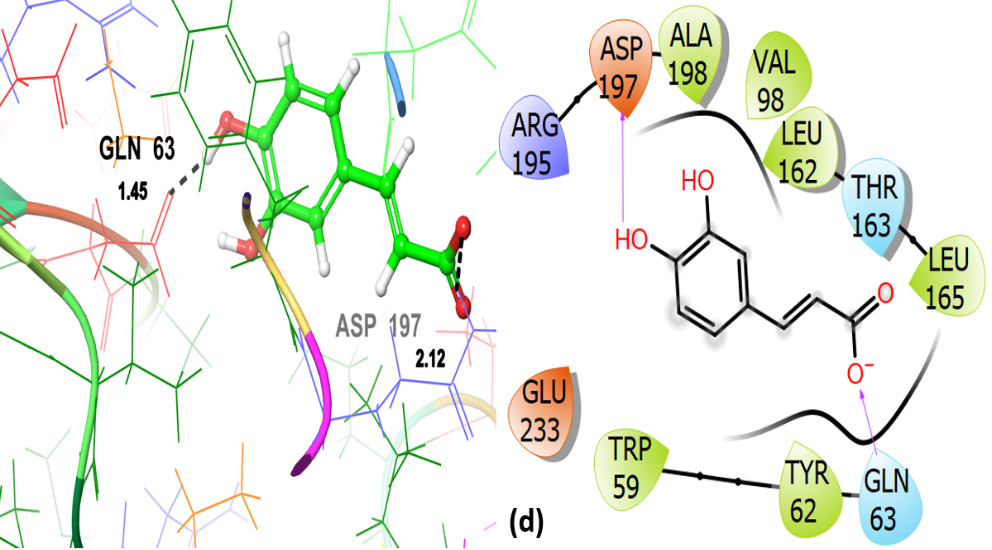


# Figure S6: Caffeic acid (d)-3d and 2d view with *α*-amylase inhibitory target protein 4W93.


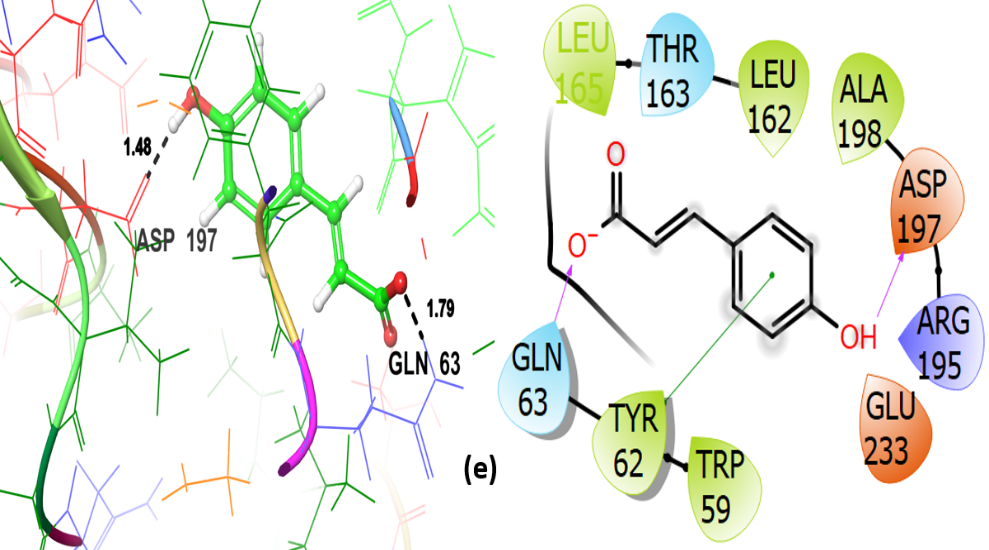


# Figure S7: *p*-coumaric acid (e)-3d and 2d view with *α*-amylase inhibitory target protein 4W93.


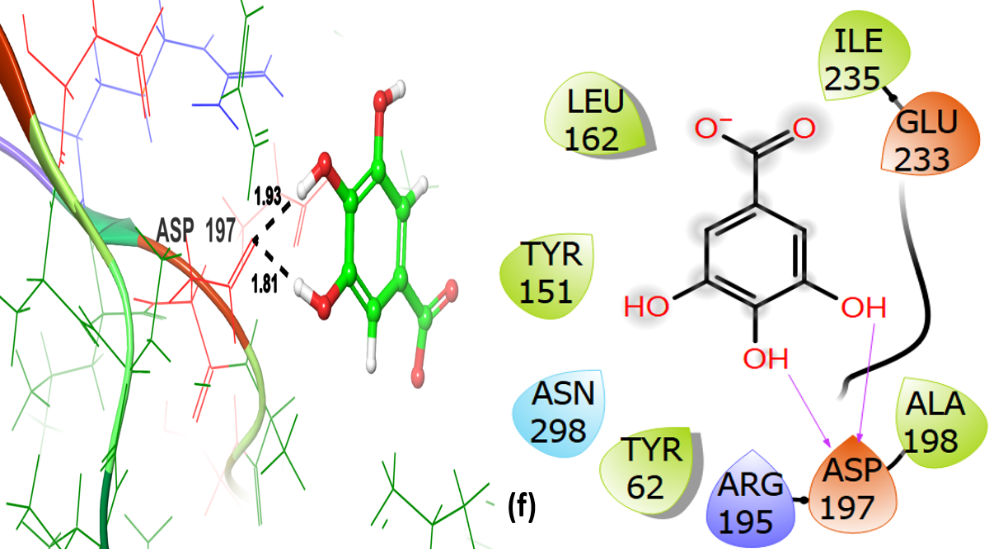


# Figure S8: Gallic acid (f)-3d and 2d view with *α*-amylase inhibitory target protein 4W93.


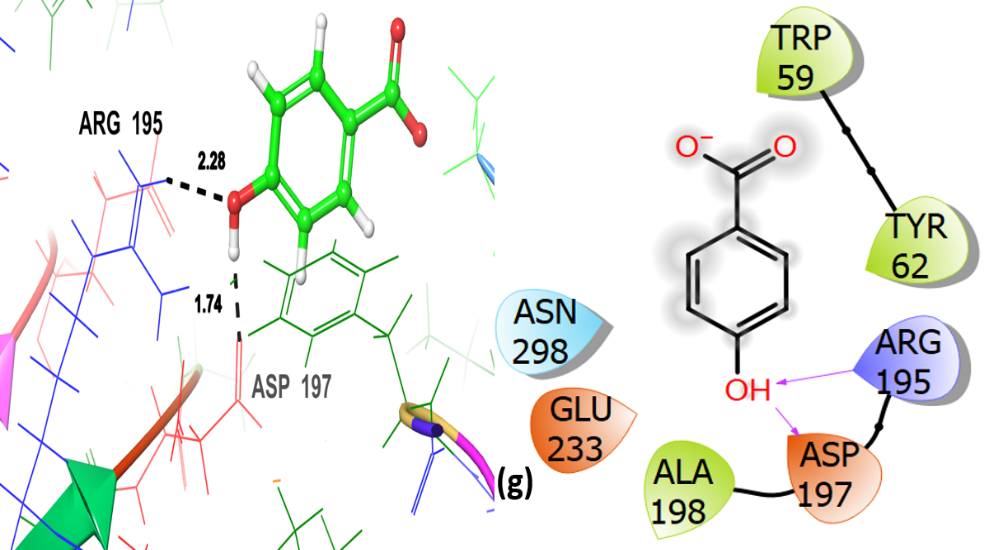


# Figure S9: Hydroxybenzoic acid (g)-3d and 2d view with *α*-amylase inhibitory target protein 4W93.


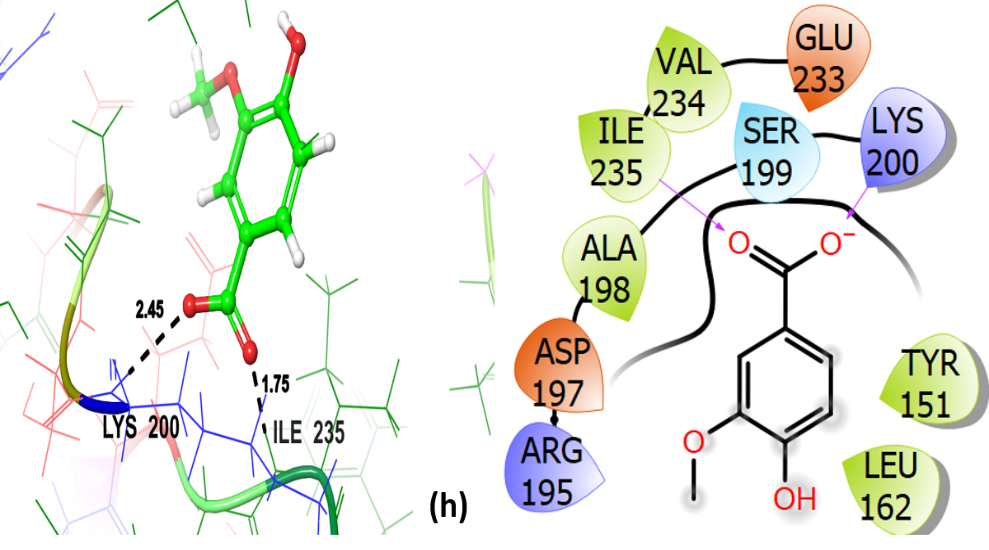


# Figure S10: Vanillic acid (h)-3d and 2d view with *α*-amylase inhibitory target protein 4W93.
